# Calciprotein Particles Link Disturbed Mineral Homeostasis with Cardiovascular Disease by Causing Endothelial Dysfunction and Vascular Inflammation

**DOI:** 10.3390/ijms222212458

**Published:** 2021-11-18

**Authors:** Daria K. Shishkova, Elena A. Velikanova, Leo A. Bogdanov, Maxim Yu. Sinitsky, Alexander E. Kostyunin, Anna V. Tsepokina, Olga V. Gruzdeva, Andrey V. Mironov, Rinat A. Mukhamadiyarov, Tatiana V. Glushkova, Evgenia O. Krivkina, Vera G. Matveeva, Oksana N. Hryachkova, Victoria E. Markova, Yulia A. Dyleva, Ekaterina V. Belik, Alexey V. Frolov, Amin R. Shabaev, Olga S. Efimova, Anna N. Popova, Valentina Yu. Malysheva, Roman P. Kolmykov, Oleg G. Sevostyanov, Dmitriy M. Russakov, Viatcheslav F. Dolganyuk, Anton K. Gutakovsky, Yuriy A. Zhivodkov, Anton S. Kozhukhov, Elena B. Brusina, Zinfer R. Ismagilov, Olga L. Barbarash, Arseniy E. Yuzhalin, Anton G. Kutikhin

**Affiliations:** 1 Research Institute for Complex Issues of Cardiovascular Diseases, 6 Sosnovy Boulevard, 650002 Kemerovo, Russia; shidk@kemcardio.ru (D.K.S.); veliea@kemcardio.ru (E.A.V.); bogdla@kemcardio.ru (L.A.B.); sinimy@kemcardio.ru (M.Y.S.); kostae@kemcardio.ru (A.E.K.); tsepav@kemcardio.ru (A.V.T.); gruzov@kemcardio.ru (O.V.G.); miroav@kemcardio.ru (A.V.M.); mukhra@kemcardio.ru (R.A.M.); glushtv@kemcardio.ru (T.V.G.); kriveo@kemcardio.ru (E.O.K.); matvvg@kemcardio.ru (V.G.M.); hryaon@kemcardio.ru (O.N.H.); markve@kemcardio.ru (V.E.M.); dyleya@kemcardio.ru (Y.A.D.); believ@kemcardio.ru (E.V.B.); frolav@kemcardio.ru (A.V.F.); shabar@kemcardio.ru (A.R.S.); brusina.eb@kemsma.ru (E.B.B.); barbol@kemcardio.ru (O.L.B.); yuzhae@kemcardio.ru (A.E.Y.); 2 Institute of Coal Chemistry and Material Science, Federal Research Center of Coal and Coal Chemistry, Siberian Branch of the Russian Academy of Sciences, 18 Sovetskiy Avenue, 650000 Kemerovo, Russia; efimovaos@iccms.sbras.ru (O.S.E.); popovaan@iccms.sbras.ru (A.N.P.); malyshevavyu@iccms.sbras.ru (V.Y.M.); kolmykovrp@iccms.sbras.ru (R.P.K.); ismagilovzr@iccms.sbras.ru (Z.R.I.); 3 Institute of Fundamental Sciences, Kemerovo State University, 6 Krasnaya Street, 650000 Kemerovo, Russia; sevostyanov@kemsu.ru (O.G.S.); russakov@kemsu.ru (D.M.R.); dolganyuk@kemsu.ru (V.F.D.); 4 Rzhanov Institute of Semiconductor Physics, Siberian Branch of the Russian Academy of Sciences, 13 Akademika Lavrentieva Avenue, 630090 Novosibirsk, Russia; gut@isp.nsc.ru (A.K.G.); zhivodkov@isp.nsc.ru (Y.A.Z.); kozhukhov@isp.nsc.ru (A.S.K.)

**Keywords:** calciprotein particles, endothelial dysfunction, intimal hyperplasia, vascular inflammation, cardiovascular disease

## Abstract

An association between high serum calcium/phosphate and cardiovascular events or death is well-established. However, a mechanistic explanation of this correlation is lacking. Here, we examined the role of calciprotein particles (CPPs), nanoscale bodies forming in the human blood upon its supersaturation with calcium and phosphate, in cardiovascular disease. The serum of patients with coronary artery disease or cerebrovascular disease displayed an increased propensity to form CPPs in combination with elevated ionised calcium as well as reduced albumin levels, altogether indicative of reduced Ca^2+^-binding capacity. Intravenous administration of CPPs to normolipidemic and normotensive Wistar rats provoked intimal hyperplasia and adventitial/perivascular inflammation in both balloon-injured and intact aortas in the absence of other cardiovascular risk factors. Upon the addition to primary human arterial endothelial cells, CPPs induced lysosome-dependent cell death, promoted the release of pro-inflammatory cytokines, stimulated leukocyte adhesion, and triggered endothelial-to-mesenchymal transition. We concluded that CPPs, which are formed in the blood as a result of altered mineral homeostasis, cause endothelial dysfunction and vascular inflammation, thereby contributing to the development of cardiovascular disease.

## 1. Introduction

A correlation between elevated serum calcium/phosphate levels and coronary artery disease [1,2], stroke [2,3], and cardiovascular death [2,3,4] has been long recognised, yet the mechanism behind this link is unclear. Hyperphosphatemia, which is prevalent in patients with pre-dialysis chronic kidney disease (CKD) or end-stage renal disease (ESRD) [5], induces medial arterial calcification rather than atherosclerosis [6], thus failing to explain the aforementioned association. Structural studies on the inhibition of extraskeletal calcification uncovered the pivotal significance of the fetuin-A-driven formation of calcium phosphate nanoparticles, which mediate the clearance of excessive minerals from the circulation [7,8,9]. Recently, we proposed that such aggregation of serum calcium and phosphate into calciprotein particles (CPPs) could, in principle, provoke endothelial dysfunction and adventitial/perivascular inflammation [10], the pathological conditions underlying cardiovascular disease (CVD) [11].

Recent studies demonstrated higher serum propensity to generate CPPs in patients with ESRD with concomitant myocardial infarction or peripheral artery disease than in those without [12] and in CKD-free subjects suffering from arterial hypertension compared with healthy blood donors [13]. Furthermore, an elevated CPP serum level was associated with the progression of stable angina to acute coronary syndrome as well as with increased plaque volume in patients with preserved renal function [14]. Importantly, accelerated formation of CPPs in the blood correlated with an increased risk of cardiovascular events and cardiovascular death in kidney transplant recipients [15] as well as haemodialysed patients [16]. These reports highlight the clinical relevance of CPPs in the context of cardiovascular disorders. However, the mechanisms behind the detrimental impact of CPPs on arterial homeostasis remain obscure.

Here, we identified augmented CPP formation and ionised calcium along with reduced total protein and albumin, a major mineralisation inhibitor, in CKD-free patients with established coronary artery disease or cerebrovascular disease in comparison with healthy individuals. The pathogenic effects of CPPs were evident in rat models where their systemic delivery induced intimal hyperplasia and adventitial/perivascular inflammation in balloon-injured and intact aortas without any confounding cardiovascular risk factors. Further mechanistic insight into the CPP-driven vascular injury revealed that CPPs promote the lysosome-dependent death of endothelial cells (ECs), endothelial activation, and endothelial-to-mesenchymal transition. In accordance with molecularly oriented definitions of cell death subroutines [17], the lysosome-dependent cell death pathway, triggered by internalisation of CPPs by ECs and distribution of internalised CPPs to lysosomes, involves primary lysosome membrane permeabilisation because of increased lysosomal Ca^2+^ content after CPP dissolution, disrupted osmotic balance between the lysosomes and cytosol, Ca^2+^ influx into the cytosol, and activation (cleavage) of executioner caspases such as caspase-3 and its substrates. Collectively, these findings underscore the pathophysiological importance of decreased serum albumin along with augmented ionised calcium (Ca^2+^), pinpoint increased ionised calcium as a chemical substrate for elevated serum propensity to form CPPs, and implicate CPPs as a probable mechanistic link between disturbed mineral homeostasis and CVD.

## 2. Results

### 2.1. Atherosclerotic Vascular Disease Is Characterised by Augmented CPP Formation as a Consequence of Disturbed Mineral Homeostasis

**Figure 1:**
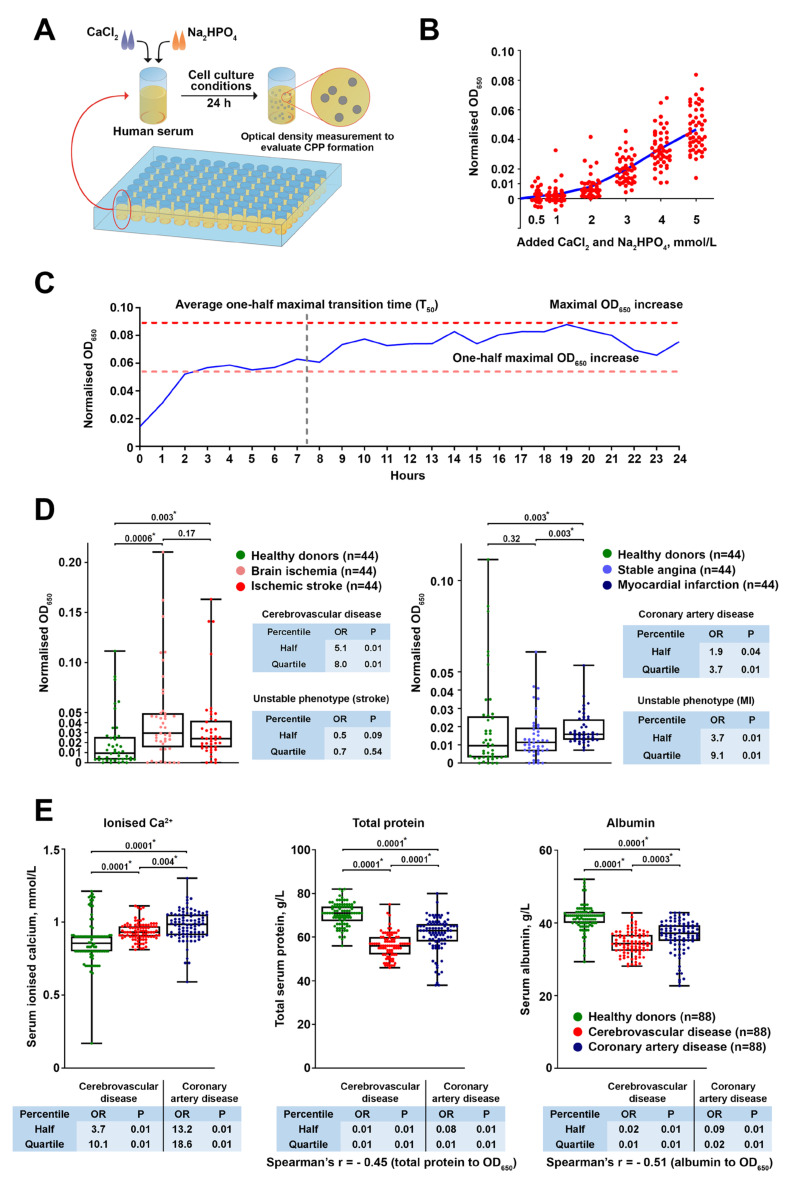
Generation of CPPs in the blood is a distinctive feature of cerebrovascular disease and coronary artery disease. (**A**) Experimental pipeline assaying human serum propensity to produce CPPs. The assay includes addition of excessive CaCl_2_ and Na_2_HPO_4_ concentrations to the serum followed by 24 h incubation in cell culture conditions and measurement of the optical density at a wavelength of 650 nm (OD_650_), followed by background subtraction (OD_650_ of the serum sample without added CaCl_2_ and Na_2_HPO_4_). (**B**) Determination of the optimal amount of added CaCl_2_ and Na_2_HPO_4_ for the assay described in (**A**). Ascending equimolar concentrations (0.5, 1, 2, 3, 4, and 5 mmol/L) of CaCl_2_ and Na_2_HPO_4_ were added to the serum of healthy individuals (n = 49) followed by OD_650_ measurement and background subtraction. The 2 mmol/L of added CaCl_2_ and Na_2_HPO_4_ was a threshold above which all samples showed an OD_650_ > 0 after the background subtraction, which indicated CPP formation. (**C**) Temporal pattern of CPP formation in the setup from (**A**). Equimolar (2 mmol/L) concentrations of CaCl_2_ and Na_2_HPO_4_ were added to the serum of healthy individuals (n = 42) with the subsequent time-lapse measurement of OD_650_ to evaluate CPP formation over time. The average one-half maximal transition time (T_50_) reflects the average time required for reaching one-half of the maximal OD_650_ increase across the samples. The one-half maximal OD_650_ increase reflects the average OD_650_ value equal to half of the maximal OD_650_ increase across the samples. (**D**) Equimolar (2 mmol/L) concentrations of CaCl_2_ and Na_2_HPO_4_ were added to the serum of healthy individuals (n = 44) and patients with brain ischemia that required carotid endarterectomy (n = 44), ischemic stroke (n = 44), stable angina that required coronary artery bypass graft surgery (n = 44), or myocardial infarction (n = 44), followed by OD_650_ measurement and background subtraction. Note an increase in the CPP formation in the brain ischemia, ischemic stroke, and myocardial infarction groups. The tables to the right represent the odds ratios and *p*-values for the risk of cerebrovascular disease, coronary artery disease, or unstable plaque phenotype in percentiles with the highest and lowest OD_650_ increases. (**E**) Measurement of ionised calcium (Ca^2+^), total protein, and albumin levels in the serum of healthy individuals (n = 88) and patients with cerebrovascular disease (brain ischemia and ischemic stroke combined, n = 88) or coronary artery disease (stable angina and myocardial infarction combined, n = 88). The tables below represent the odds ratios and *p*-values for the risk of cerebrovascular disease or coronary artery disease in percentiles with the highest and lowest Ca^2+^, total protein, and albumin values. Each dot represents a serum sample collected from one subject. Whiskers indicate ranges, box bounds indicate the 25th–75th percentiles, centre lines indicate the median. *p*-values are provided above the boxes (statistically significant differences are marked by asterisks), Kruskal–Wallis test with post hoc false discovery rate correction by two-stage linear step-up procedure of Benjamini, Krieger, and Yekutieli. CPP—calciprotein particles, OD_650_—optical density at 650 nm wavelength.

To measure the ability of human serum to generate CPPs, we set up an assay in which excessive concentrations of CaCl_2_ and Na_2_HPO_4_ were added to the serum with subsequent 24 h incubation in cell culture conditions to maintain temperature, humidity, and pH during CPP production (Figure 1A). The 2 mmol/L of added calcium salts and phosphates was a threshold above which all the healthy blood donors showed an elevation in optical density, indicative of CPP formation [9,10,18,19] (Figure 1B). Time-lapse monitoring of CPP formation documented an initial sharp rise in the optical density, followed by a gradual increase, reaching its maximum at the end of the incubation (Figure 1C), thereby indicating 24 h as the time point of serum saturation with CPPs. We then aimed to examine whether patients with established CVD are more prone to serum CPP generation than asymptomatic subjects.

**Figure 2:**
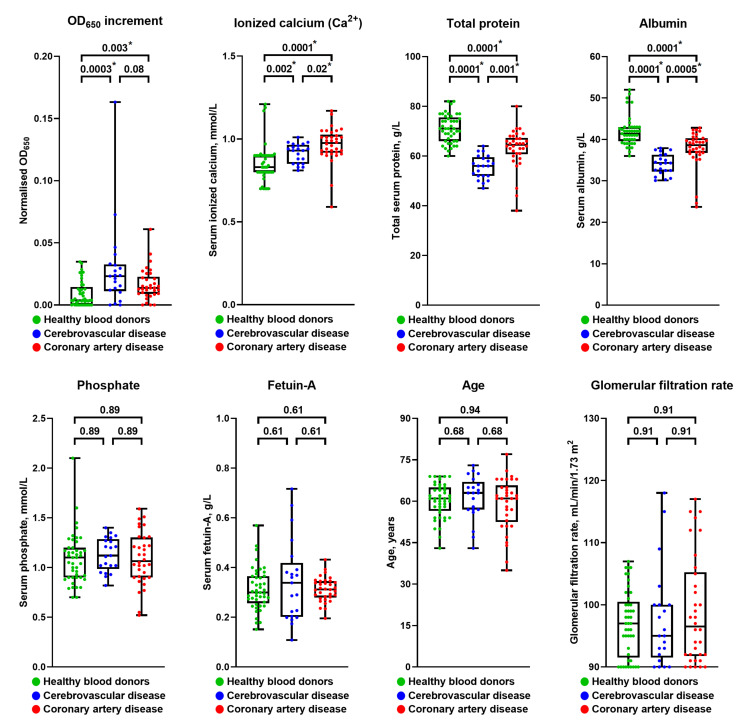
Estimated glomerular filtration rate does not define serum CPP generation propensity. Filtration of the subjects in Figure 1 by eGFR (selection of those with eGFR ≥ 90 mL/min/m^2^ for the subsequent analysis) and repeated statistical analysis of OD_650_ increase (CPP generation propensity), serum ionised calcium (Ca^2+^), total protein and albumin concentration, phosphate and fetuin-A level, and age. Note that the results of this repeated statistical analysis do not differ from those without eGFR stratification, i.e., increased CPP generation propensity and serum ionised calcium along with reduced total protein and albumin levels in patients with cerebrovascular disease and coronary artery disease. Note the absence of statistically significant differences in serum phosphate and fetuin-A levels as well as subject age, similar to the general cohorts. Each dot represents a serum sample collected from one subject. Whiskers indicate range, box bounds indicate 25th–75th percentiles, and centre lines indicate median. *p*-values are provided above boxes (statistically significant differences are marked by asterisks), Kruskal–Wallis test with post hoc false discovery rate correction by two-stage linear step-up procedure of Benjamini, Krieger, and Yekutieli. CPP—calciprotein particles, eGFR—estimated glomerular filtration rate, and OD_650_—optical density at 650 nm wavelength.

The serum of patients with cerebrovascular disease and myocardial infarction exhibited a significantly higher propensity to form CPPs in comparison with healthy volunteers (Figure 1D). Strikingly, individuals with the highest CPP yield had a considerably higher risk of carotid and coronary atherosclerotic disease as well as unstable coronary plaque phenotype compared with those having the lowest amount of CPPs (Figure 1D). To further explore the mechanism behind the pronounced CPP formation in the serum of CVD patients, we comprehensively evaluated their mineral homeostasis. Patients with cerebrovascular disease or coronary artery disease had higher levels of ionised calcium (Ca^2+^) along with reduced total protein and albumin (Figure 1E), collectively suggestive of exhausted serum Ca^2+^-binding capacity. Importantly, the serum levels of albumin and total protein (60% comprised of albumin) inversely correlated with optical density increases, suggesting albumin’s regulatory role in CPP formation, which surprisingly was unaffected by serum phosphate or fetuin-A concentrations (Figure S1 and Table S1). The clinicopathological features of the study participants are presented in Table S2. The restriction of subject cohorts to the subjects having estimated glomerular filtration rate (eGFR) > 90 mL/min/1.73 m^2^ did not change the results (Figure 2), suggesting that eGFR does not define CPP-generation propensity. After repeating statistical analysis in healthy volunteers and patients with cerebrovascular or coronary artery disease with eGFR > 90 mL/min/1.73 m^2^, the patient cohorts still demonstrated higher CPP generation propensity, increased serum ionised calcium, and reduced total protein and albumin levels (Figure 2), suggesting that eGFR is not a major factor affecting CPP formation.

Taken together, these findings demonstrate that atherosclerotic vascular disease is frequently accompanied by disturbed mineral homeostasis, resulting in enhanced aggregation of serum Ca^2+^ and PO_4_^3−^ into the CPPs. Hence, we propose that CPP generation is potentially involved in the development of CVD.

### 2.2. Recapitulation of the Physiological Scenario by Synthesising Primary and Secondary CPPs

**Figure 3:**
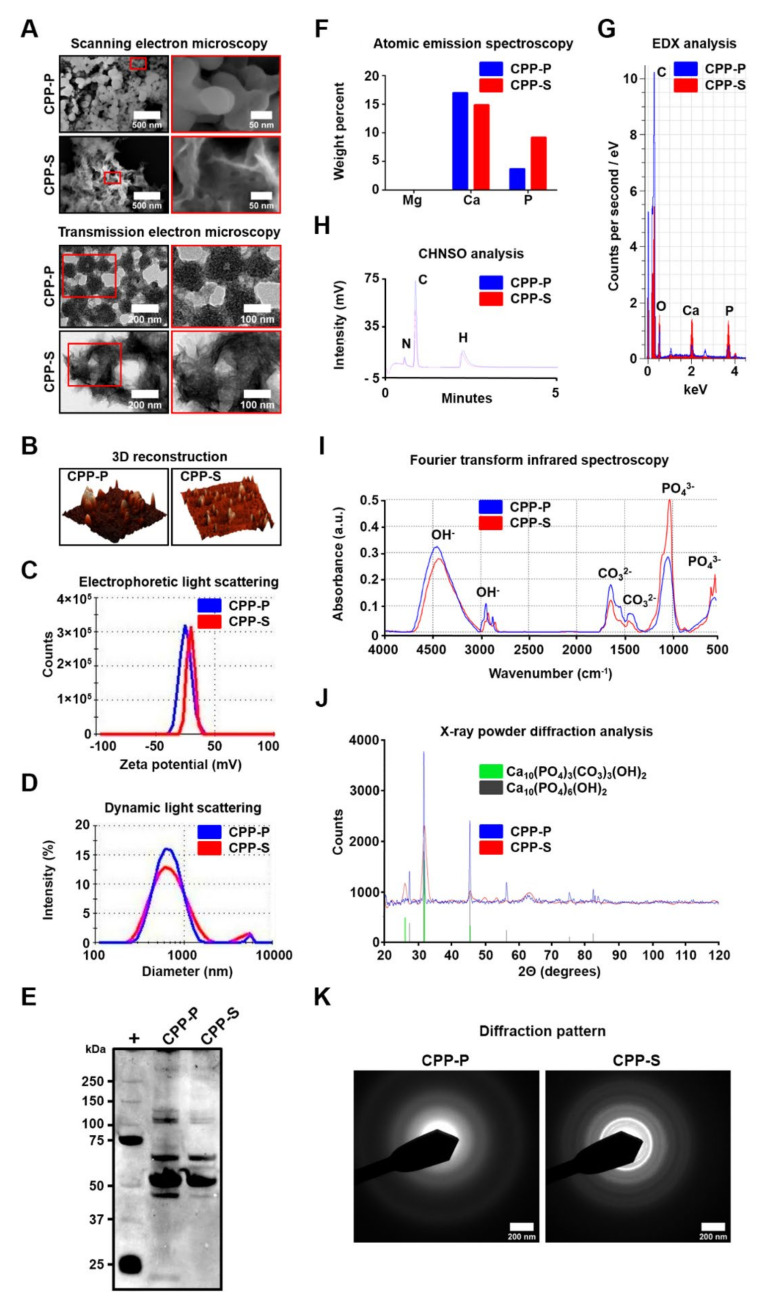
In vitro synthesised CPP-P are amorphous and round, whereas CPP-S represent crystalline, spindle-shaped nanoparticles with a higher mineral content. (**A**) Scanning and transmission electron microscopy images showing that CPP-P are spherical particles whereas CPP-S have a spindle- or needle-like shape; (**B**) 3D reconstruction of CPP-P and CPP-S by atomic force microscopy. (**C**) Electrophoretic light scattering plot showing that CPP-P and CPP-S have a nearly identical surface charge (from −20 to −25 mV) indicative of their capability to aggregate. (**D**) Dynamic light scattering measurements demonstrate that CPP-P and CPP-S have a similar particle-size distribution curve, with a diameter range of 100–1000 nm and average diameter of around 500 nm. (**E**) Sodium dodecyl sulphate-polyacrylamide gel electrophoresis with subsequent silver staining show that CPP-P and CPP-S have a similar protein profile. (**F**) Atomic emission spectroscopy confirms that both CPP-P and CPP-S contain calcium and phosphorus. (**G**) Energy-dispersive X-ray spectroscopy shows higher carbon content in CPP-P but an increased proportion of calcium and phosphorus in CPP-S. (**H**) CHNSO analysis allows detection of nitrogen, carbon, and hydrogen in both CPP-P and CPP-S, also documenting higher amounts of carbon in CPP-P than in CPP-S. (**I**) Fourier transform infrared spectroscopy identifies phosphate (PO_4_^3−^), carbonate (CO_3_^2−^), and hydroxyl (OH^−^) groups in both CPP-P and CPP-S. Note the higher phosphate content in CPP-S compared with CPP-P. (**J**) X-ray powder diffraction analysis demonstrates that both CPP-P and CPP-S consist of hydroxyapatite (Ca_10_(PO_4_)_6_(OH)_2_ and carbonate-hydroxyapatite (Ca_10_(PO_4_)_3_(CO_3_)_3_(OH)_2_). (**K**) Selected area diffraction patterns indicate the lower crystallinity of CPP-P in comparison with CPP-S. CPP-P—primary calciprotein particles, CPP-S—secondary calciprotein particles, and EDX—energy-dispersive X-ray spectroscopy.

The noticeable abrupt increase in serum optical density within 2 h of incubation supported the amorphous-to-crystalline transition, a phenomenon [19] reflecting the transformation of primary CPPs, which are relatively amorphous and round, into secondary crystalline spindle-shaped particles (Figure 3A,B). Although primary CPPs are generally considered harmless, in contrast to secondary CPPs possessing deleterious effects [20], recent findings summarised in a critical review challenged this hypothesis [21]. In this study, we aimed to examine the pathogenic effects of both CPP types as the internalisation of CPP-P might occur within 1 h of circulation [22,23]; therefore, both CPP-P and CPP-S can be internalised by endothelial cells, though in variable amounts, in a physiological scenario.

To reproduce the formation of both primary and secondary CPPs (CPP-P and CPP-S, respectively) in human serum, we used an established technique of in vitro nanoparticle synthesis involving the sequential addition of CaCl_2_ and Na_2_HPO_4_ to the serum-containing culture medium, followed by incubation in cell culture conditions for 24 h [9,10]. Previous findings demonstrated that the size, shape, structure, and chemical profile of plaque-derived and artificially synthesised CPPs are identical [10], providing a rationale for using the latter in experimental settings.

As detrimental effects of nanoparticles are largely defined by their physical and chemical properties, we applied a nanomaterial approach to comprehensively characterise CPP-P and CPP-S. Th surface charges of both particle types were similar, ranging from −20 to −25 mV (Figure 3C), suggestive of their capability to aggregate; this was corroborated by dynamic light scattering analysis detecting clusters of up to 1000 nm in diameter (Figure 3D). CPP-P and CPP-S had similar composition of associated proteins, which were reported to abate the cytotoxicity of mineral particles [24] (Figure 3E). Regarding the mineral profile, both CPP-P and CPP-S contained significant amounts of calcium, phosphate, carbon, oxygen, and hydrogen (Figure 3F–H). Yet, pronounced carbon peaks were notable in CPP-P, whereas mineral content was generally higher in CPP-S (Figure 3F–H). The mentioned chemical elements formed phosphate (PO_4_^3−^), carbonate (CO_3_^2−^), and hydroxyl (OH^−^) functional groups (Figure 3I), together constituting hydroxyapatite and carbonate-hydroxyapatite, the ultimate products of calcium phosphate maturation (Figure 3J). The diffraction pattern of CPP-P indicated their lower crystallinity in comparison with CPP-S (Figure 3K).

Similarly, we also synthesised magnesiprotein particles (MPPs) by replacing CaCl_2_ with MgCl_2_ in order to determine whether deleterious putative CPP-driven effects can be inflicted by particles containing other cations. Similar to CPPs, MPPs appeared as spherical nanoparticles that had an identical surface charge, particle-size distribution, and protein profile, but contained magnesium with a negligible level of calcium (Figure S2).

In addition, we performed a characterisation of the CPPs generated in the serum from the CPP formation assay (addition of 2 mmol/L CaCl_2_ and Na_2_HPO_4_ to the human serum followed by incubation at 37 °C, 5% CO_2_: 95% air, and high humidity for 24 h). Upon the centrifugation of the diluted serum at 200,000× *g* and delipidation for dissolving the extracellular vesicles, we obtained a clear white sediment with a pronounced calcium and phosphate content and appearance similar to artificially synthesised CPP-P (Figure S3).

### 2.3. CPPs Provoke Intimal Hyperplasia and Vascular Inflammation in the Absence of Other Cardiovascular Risk Factors

**Figure 4:**
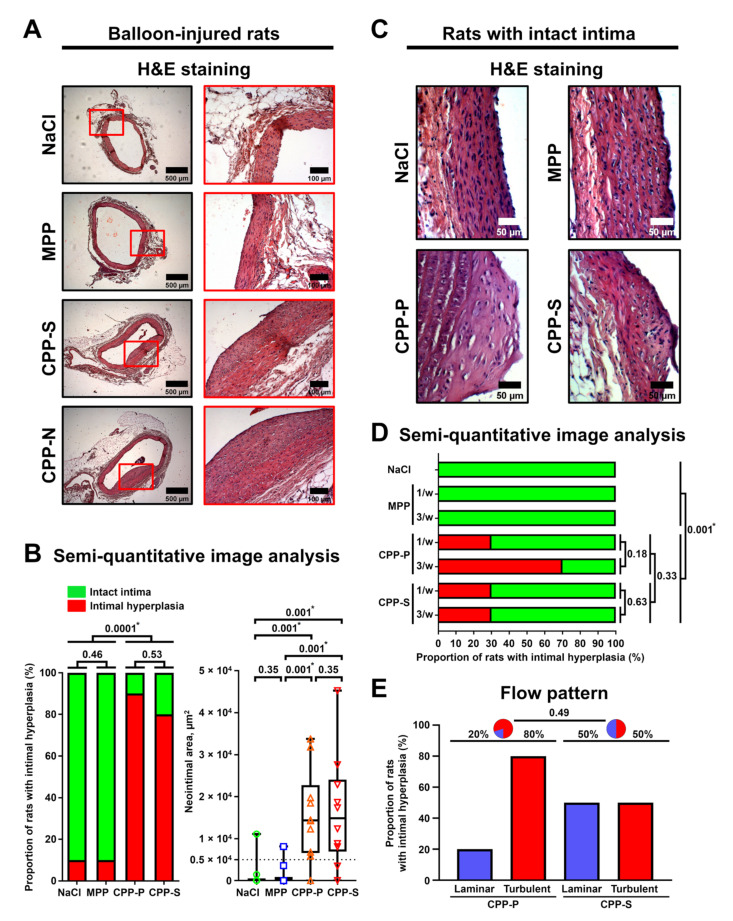
CPPs induce intimal hyperplasia in both injured and intact rat aortas. (**A**) H&E-stained aortas from balloon-injured rats treated by a single intravenous injection of NaCl, MPPs, CPP-P, or CPP-S (900 μL of particles, OD_650_ = 0.08–0.10, for CPP-P and CPP-S: equal to 450 μg calcium or 1.08 × 10^6^ OsteoSense 680EX-positive PKH67-negative events) immediately postoperation (n = 10 rats per group, 40 rats in total). Note the intimal hyperplasia in rats treated with CPP-P and CPP-S. (**B**) Proportion of rats with intimal hyperplasia and neointimal area calculated upon the analysis of H&E-stained aortas. Each dot represents an aortic section from one rat. Note that the zero neointimal area corresponds to the absence of neointima. The dotted line indicates a threshold value for the definition of intimal hyperplasia (≥0.5 × 10^4^ μm^2^). Whiskers indicate range, box bounds indicate the 25th–75th percentiles, and centre lines indicate the median. *p*-values are provided above boxes (statistically significant differences are marked by asterisks), Pearson’s chi-squared test with Yates’s correction for continuity or Kruskal–Wallis test with post hoc false discovery rate correction by two-stage linear step-up procedure of Benjamini, Krieger, and Yekutieli. (**C**) H&E-stained aortas from rats intravenously injected with NaCl, MPPs, CPP-P, or CPP-S (900 μL of particles per injection, OD_650_ = 0.08–0.10, for CPP-P and CPP-S: equal to 450 μg calcium or 1.08 × 10^6^ OsteoSense 680EX-positive PKH67-negative events) once or thrice a week (n = 10 rats per group, 70 rats in total) for a total duration of 5 weeks. Note the intimal hyperplasia in rats treated with CPP-P and CPP-S. (**D**) Semi-quantitative image analysis of H&E-stained aortas of rats from the experiment in (**C**). Shown is the proportion of rats with intimal hyperplasia. Pearson’s chi-squared test with Yates’s correction for continuity. (**E**) Association of CPP-induced intimal hyperplasia with flow patterns characteristic of different aortic segments (aortic arch with a turbulent flow and descending aorta characterised by a laminar flow). Shown is the proportion of rats with intimal hyperplasia. Pie charts represent the proportions of laminar and turbulent segments (i.e., descending aorta and aortic arch, respectively) among those with intimal hyperplasia upon CPP-P or CPP-S administration. Pearson’s chi-squared test with Yates’s correction for continuity. H&E—haematoxylin and eosin, MPPs—magnesiprotein particles, CPP-P—primary calciprotein particles, CPP-S—secondary calciprotein particles, and OD_650_—optical density at 650 nm wavelength.

To test if CPPs are able to significantly impair vascular homeostasis, we investigated whether these particles can induce atherosclerosis-associated lesions in wild-type Wistar rats free of known cardiovascular risk factors. Even a single intravenous injection of CPP-P or CPP-S immediately after balloon-induced aortic injury led to the development of intimal hyperplasia (Figure 4A) in 90% and 80% of animals 5 weeks postoperation, respectively, indicating that CPPs may aggravate pre-existing vascular injury (Figure 4B). The administration of MPPs rarely (10% of rats) caused neointima formation (Figure 4B). To challenge the ability of CPPs to cause intimal hyperplasia per se, we administered CPPs once or thrice a week over 5 weeks to rats that did not undergo surgical intervention. We observed intimal hyperplasia (Figure 4C) in 50% of rats that received CPP-P and 30% of rats injected with CPP-S without a clear dose-dependency (Figure 4D). Consistent with the angioplasty model, administration of MPPs did not result in notable intimal lesions (Figure 4D). CPP-P provoked intimal hyperplasia primarily in the aortic arch, characterised by a turbulent flow, whereas no correlation between aortic lesions and flow pattern was found in animals infused with CPP-S, though this difference did not reach a statistical significance (Figure 4E). Importantly, no signs of vascular calcification were observed regardless of animal model or CPP administration regimen (Figure S4). Collectively, these results indicate the capability of CPPs to trigger neointima formation in a normolipidemic and normotensive animal model.

**Figure 5:**
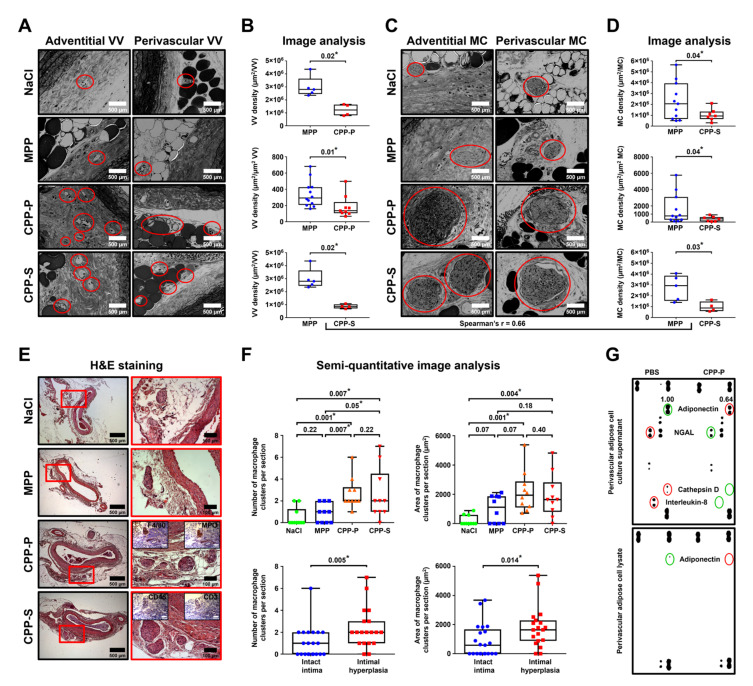
CPPs induce adventitial and perivascular inflammation which correlates with intimal hyperplasia. (**A**) Representative images of adventitial and perivascular vasa vasorum (VV) in the aortas of rats that underwent balloon angioplasty followed by daily intravenous injections of NaCl, MPPs, CPP-P, or CPP-S (900 μL of particles per injection, OD_650_ = 0.08–0.10, for CPP-P and CPP-S: equal to 450 μg calcium or 1.08 × 10^6^ OsteoSense 680EX-positive PKH67-negative events) for 5 days (n = 5 rats per group, 20 rats in total). Rats were euthanised 5 weeks postoperation with subsequent backscattered scanning electron microscopy examination of the injured (n = 7–12) and intact (n = 4–5) aortic segments. Red circles denote areas with VV. (**B**) Semi-quantitative image analysis of the VV from the experiment in (**A**). VV density was calculated for both VV number and VV area (area of the adventitial and perivascular adipose tissue area per one vessel (μm^2^) or 1 μm^2^ of the VV area, respectively). (**C**) Representative images of adventitial and perivascular macrophage clusters (MCs) in the rat aortas from the experiment in (**A**). Red circles denote MCs. (**D**) Semi-quantitative image analysis of the MCs from the experiment in (**C**). MC density was calculated in similar to VV density. (**E**) H&E-stained aortas containing MCs from balloon-injured rats treated by a single intravenous injection of NaCl, MPP, CPP-P, or CPP-S immediately postoperation (n = 10 rats per group, 40 rats in total). Red quadrants within the whole section images (left) indicate regions of interest with MCs (right). Insets confirm positive staining of MCs for a macrophage marker F4/80 and myeloperoxidase (MPO), an enzyme reflecting macrophage activity. MCs are also negative for T cell marker CD3. (**F**) Semi-quantitative image analysis of the MCs in the rat aortas from the experiment in €. Each dot represents an aortic section from one rat. Whiskers indicate the range, box bounds indicate the 25th–75th percentiles, and centre lines indicate the median. *p*-values are provided above boxes (statistically significant differences are marked by asterisks), Kruskal–Wallis test with post hoc false discovery rate correction by two-stage linear step-up procedure of Benjamini, Krieger, and Yekutieli or Mann–Whitney U-test. (**G**) Primary human perivascular adipocytes were treated with PBS or CPP-P (50 μL of particles per well on a 24-well plate, OD_650_ = 0.08–0.10, equal to 25 μg calcium or 0.6 × 10^5^ OsteoSense 680EX-positive PKH67-negative events) for 24 h. Conditioned media and cell lysate were profiled for adipokines and cytokines. Numbers indicate densitometry relative to PBS group. Red and green circles indicate increase and decrease in the adipokine/cytokine level, respectively. Note a decrease in adiponectin in CPP-P-treated cells. VV—vasa vasorum, MPPs—magnesiprotein particles, CPP-P—primary calciprotein particles, CPP-S—secondary calciprotein particles, MCs—macrophage clusters, H&E—haematoxylin and eosin, MPO—myeloperoxidase, and PBS—phosphate-buffered saline.

Several lines of evidence show that adventitial and perivascular inflammation contributes to the development of atherosclerosis [25]. To investigate whether CPPs affect blood vessels in this regard, we employed another animal model where Wistar rats underwent daily intravenous injections of MPPs, CPP-P, or CPP-S during the first week upon balloon angioplasty and were euthanised 5 weeks postoperation, with subsequent electron microscopy visualisation of the aortas. We then examined the vasa vasorum (VV) and macrophage clusters (MCs), which considerably expand during the course of adventitial/perivascular inflammation [26]. The aortas of rats that received CPP-P or CPP-S displayed an increased density of both VV (Figure 5A,B) and MCs (Figure 5C,D) within the adventitia and perivascular adipose tissue compared to those of MPP-treated animals in both balloon-injured and intact aortic segments. Importantly, the number of VV and MCs well-correlated to each other (Spearman’s r = 0.66). To verify these results, we quantified the MCs in rat aortas from the initial angioplasty model by routine histological examination (Figure 5E). The area and number of MCs were significantly higher in rats injected with CPP-P or CPP-S in comparison with mock- or MPP-treated animals and in the blood vessels with intimal hyperplasia than in those without (Figure 5F), confirming the proinflammatory role of MCs in CPP-induced vascular injury. Proteomic profiling of perivascular adipocytes exposed to CPPs in vitro revealed a reduced secretion of anti-angiogenic and anti-inflammatory protein adiponectin (Figure 5G), suggesting it may control CPPs’ proinflammatory effects in adventitia and perivascular adipose tissue. Intriguingly, the levels of cathepsin G and interleukin-8, which exert multiple pro-inflammatory effects, were negligible in cell culture supernatant from CPP-P-treated perivascular adipocytes (Figure 5G), implying a distinct response to CPPs by vascular cell populations.

### 2.4. Lysosome-Dependent Cell Death Defines the Toxicity of CPPs toward Endothelial Cells

**Figure 6:**
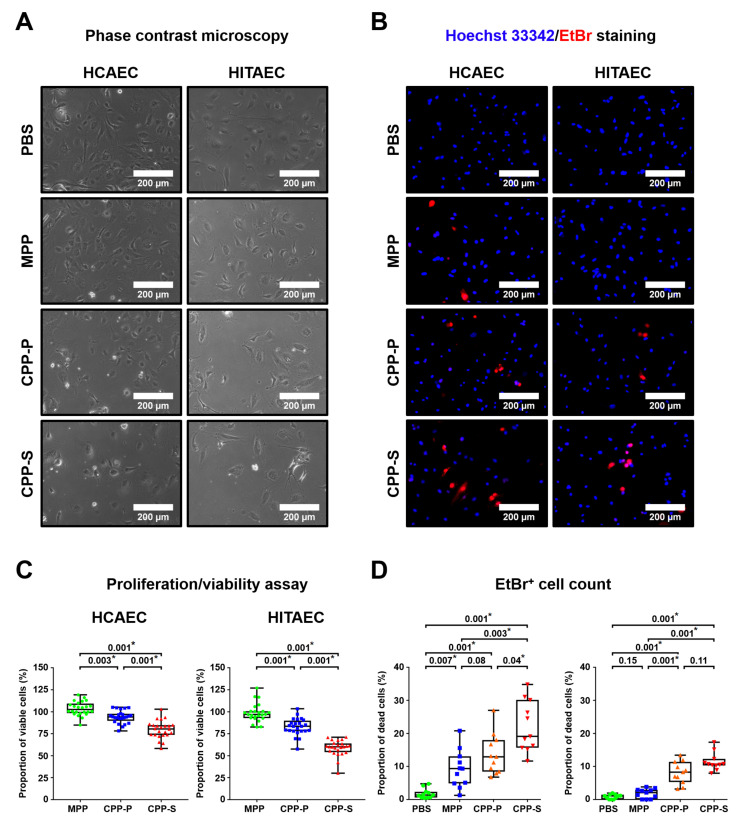
CPP-P and CPP-S inflict cell death in culture. (**A**) Phase contrast microscopy images of primary human coronary artery endothelial cells (HCAECs) and human internal thoracic artery endothelial cells (HITAECs) treated with PBS, MPPs, CPP-P, or CPP-S (100 μL of particles per well of a 6-well plate, OD_650_ = 0.08–0.10, for CPP-P and CPP-S: equal to 50 μg calcium or 1.2 × 10^5^ OsteoSense 680EX-positive PKH67-negative events) for 4 h. (**B**) Indicated cells stained for Hoechst 33342 (blue) and EtBr (red). EtBr-positive staining indicates dead cells. (**C**) Quantification of viable cells by colorimetric proliferation and viability assay after treatment with PBS, MPPs, CPP-P, or CPP-S (10 μL of particles per well of a 96-well plate, OD_650_ = 0.08–0.10, for CPP-P and CPP-S: equal to 5 μg calcium or 1.2 × 10^4^ OsteoSense 680EX-positive PKH67-negative events) for 4 h (n = 24 wells per group). The proportions of viable cells were calculated in relation to the PBS group. (**D**) Quantification of dead cells from the experiment in B (n = 11 wells per group). (**C**,**D**) Each dot represents one well of the culture plate. Whiskers indicate the range, box bounds indicate the 25th–75th percentiles, and centre lines indicate the median. *p*-values are provided above boxes (statistically significant differences are marked by asterisks), Kruskal–Wallis test with post hoc false discovery rate correction by two-stage linear step-up procedure of Benjamini, Krieger, and Yekutieli. HCAECs—human coronary artery endothelial cells, HITAECs—human internal thoracic artery endothelial cells, PBS—phosphate-buffered saline, MPPs—magnesiprotein particles, CPP-P—primary calciprotein particles, CPP-S—secondary calciprotein particles, OD_650_—optical density at 650 nm wavelength, and EtBr—ethidium bromide.

In keeping with the in vivo findings implicating CPPs in the promotion of vascular injury, the primary ECs of both atherosclerosis-susceptible human coronary artery and atherosclerosis-resistant human internal thoracic artery [27] exhibited more frequent cell death when exposed to CPP-P or CPP-S for 4 h (Figure 6A–D). In comparison with CPP-P, treatment with CPP-S was 15–25% more cytotoxic, whereas the addition of MPPs to cells led to negligible changes in proliferation and viability (Figure 6A–D).

**Figure 7:**
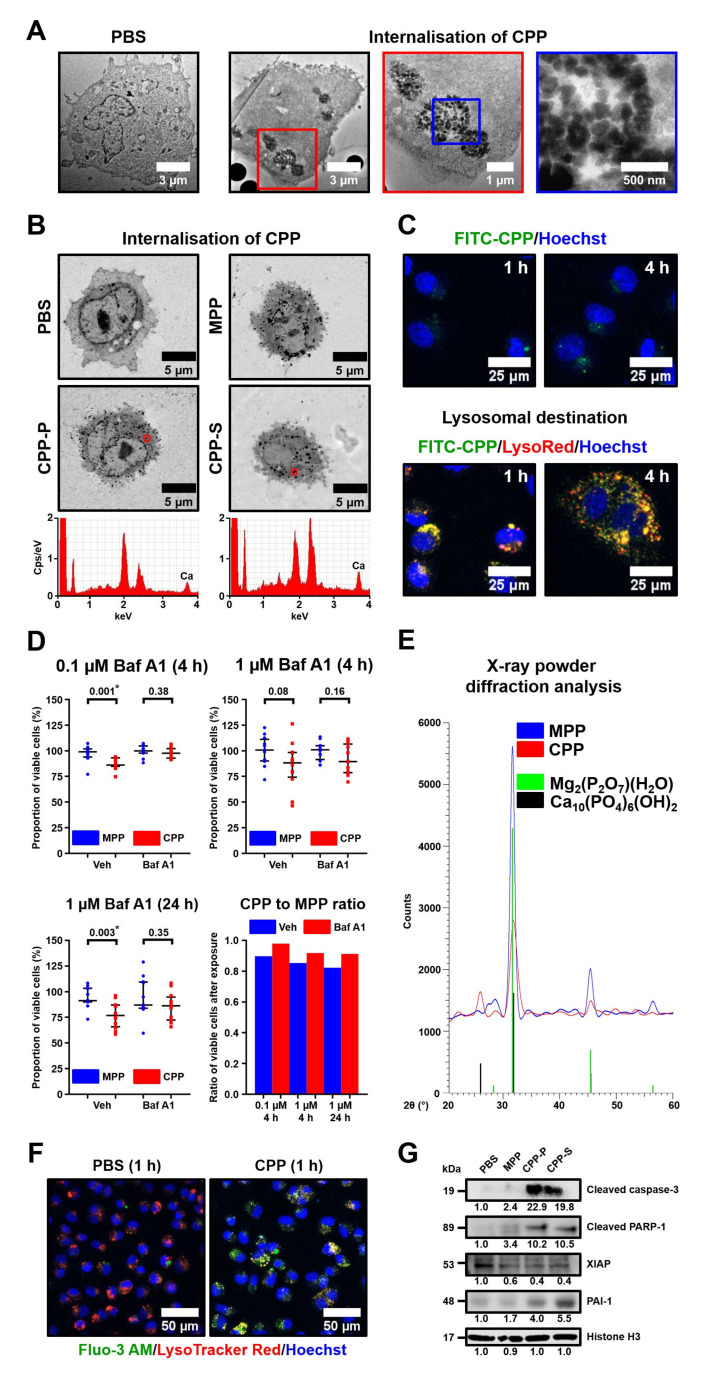
Lysosome-dependent cell death defines specific toxicity of CPPs for endothelial cells. (**A**) Transmission electron microscopy images of HCAECs treated with PBS or CPPs for 4 h. Inserts show internalised CPPs. (**B**) Backscattered scanning electron microscopy of HCAECs treated with PBS, MPPs, CPP-P, or CPP-S for 1 h. Note the electron-dense dots indicative of MPP, CPP-P, and CPP-S internalisation. Energy-dispersive X-ray spectroscopy detected Ca content when applied to these electron-dense dots in the cells treated with CPP-P or CPP-S. (**C**) Fluorescent microscopy of HCAECs treated with FITC-labelled CPPs (green) for 1 or 4 h and stained with Hoechst 33342 (nuclear stain, blue) and LysoTracker Red (lysosomes, red). Note the colocalisation of FITC-labelled CPPs with lysosomes 1 h post-treatment and partial translocation of FITC-labelled CPPs into the cytosol 4 h post-treatment. (**D**) HCAECs were treated with vehicle or ascending concentrations of bafilomycin A1 in a time-course manner and incubated in the presence of MPPs or CPPs (10 μL of particles per well of a 96-well plate, OD_650_ = 0.08–0.10, for CPPs: equal to 5 μg calcium or 1.2 × 10^4^ OsteoSense 680EX-positive PKH67-negative events) for 4 or 24 h (n = 12 wells per group) with subsequent quantitation of viable cells. Bottom-right graph demonstrates the ratio of viable cells after exposure to CPPs or MPPs. Each dot represents one well of the culture plate. Whiskers indicate the 25th–75th percentiles, and centre lines indicate the median. *p*-values provided above the graphs (statistically significant differences are marked by asterisks), Mann–Whitney U-test. (**E**) X-ray powder diffraction analysis demonstrates that MPPs and CPPs consist of highly soluble magnesium phosphate hydrate and less soluble hydroxyapatite, respectively. (**F**) HCAECs were treated with PBS or CPPs for 1 h and stained with Hoechst 33342 (nuclear stain, blue), fluo-3 AM (Ca^2+^ indicator, green), and LysoTracker Red (lysosomes, red). Note the colocalisation of Ca^2+^ with lysosomes and their partial translocation into the cytosol. (**G**) HCAECs were cultured in the presence of PBS, MPPs, CPP-P, or CPP-S (100 μL of particles per well of a 6-well plate, OD_650_ = 0.08–0.10, for CPP-P and CPP-S: equal to 50 μg calcium or 1.2 × 10^5^ OsteoSense 680EX-positive PKH67-negative events) for 4 h and immunoblotted for the indicated proteins. Histone H3 was used as the loading control. Numbers indicate densitometry relative to the PBS group. HCAECs—human coronary artery endothelial cells, PBS—phosphate-buffered saline, MPPs—magnesiprotein particles, CPP-P—primary calciprotein particles, CPP-S—secondary calciprotein particles, FITC—fluorescein isothiocyanate, OD_650_—optical density at 650 nm wavelength, Veh—vehicle control, Baf A1—bafilomycin A1, PARP-1—poly [ADP-ribose] polymerase 1, and PAI-1—plasminogen activator inhibitor 1.

Electron microscopy studies revealed the internalisation of CPPs and MPPs (Figure 7A,B) and their transport to lysosomes (Figure 7C) as early as 1 h after their addition to cultured ECs. The retained integrity of the EC membrane upon CPP internalisation suggested that CPPs could induce a specific subroutine of regulated cell death rather than causing accidental cell death. We hypothesised that the dissolution of CPPs in an acidic lysosomal milieu upon their uptake may lead to the lysosomal membrane permeabilisation triggering lysosome-dependent cell death through engaging executioner caspases. Treatment with bafilomycin A1, a compound inhibiting vacuolar-type H^+^-ATPase and therefore preventing lysosomal acidification [28] (Figure S5), partially rescued ECs from CPP-induced death (Figure 7D). Counterintuitively, MPPs consisting of magnesium phosphate hydrate (Figure 7E), which has higher solubility than CPP-specific hydroxyapatite, were unable to inflict endothelial toxicity regardless of bafilomycin A1 treatment. Hence, we speculated CPP-related EC death occurred due to the release of Ca^2+^ ions into the cytosol, which is attributable to the partial dissolution of CPPs, but not because of possible osmotic disturbance that could emerge without reference to the particle type. Staining with fluo-3 AM, a specific Ca^2+^ indicator, revealed both colocalisation of Ca^2+^ ions with lysosomes and their massive influx into the cytosol within 1 h after CPP addition to the ECs (Figure 7F), thereby supporting this hypothesis. Cleaved forms of both caspase-3 and its substrate poly [ADP-ribose] polymerase 1 (PARP-1) were elevated several fold, whereas X-linked inhibitor of apoptosis (XIAP) was reduced upon the 4 h exposure to CPP-P or CPP-S in comparison with MPP- or mock-treated cells, supporting the involvement of executioner caspases (Figure 7G). Concurrently, we detected augmented levels of plasminogen activator inhibitor 1 (PAI-1), an apoptosis inhibitor inactivating caspase-3 [29], presumably as a result of the cytoprotective stress response (Figure 7G). Taken together, these results point to lysosome-dependent cell death [17] as a probable mechanism of CPP-induced cellular demise.

Another pathway of cell death relevant for nanoparticle toxicity is respiratory burst, a phenomenon characterised by a rapid release of reactive oxygen species causing oxidative stress, which may ultimately lead to cell death via intrinsic apoptosis. The measurement of superoxide and total reactive oxygen species load using specific fluorogenic probes did not detect significant oxidative stress in ECs upon exposure to CPP-P or CPP-S, regardless of the time point (Figure S6). Quantification of thiobarbituric acid reactive substances (by-products of lipid peroxidation) in the supernatant from EC cultures also did not reveal any differences between CPP-, MPP-, and mock-treated cells (Figure S6). Along similar lines, antioxidant enzymes superoxide dismutase and catalase did not rescue ECs from CPP-induced death (Figure S6). Therefore, we suggest lysosomes but not reactive oxygen species producing organelles (i.e., mitochondria, peroxisomes, endoplasmic reticulum, and cell membrane) as the cell compartment primarily damaged upon CPP internalisation, although cytosol and mitochondria are later involved in lysosome-dependent cell death.

### 2.5. CPPs Cause Endothelial Activation and Endothelial-to-Mesenchymal Transition

**Figure 8:**
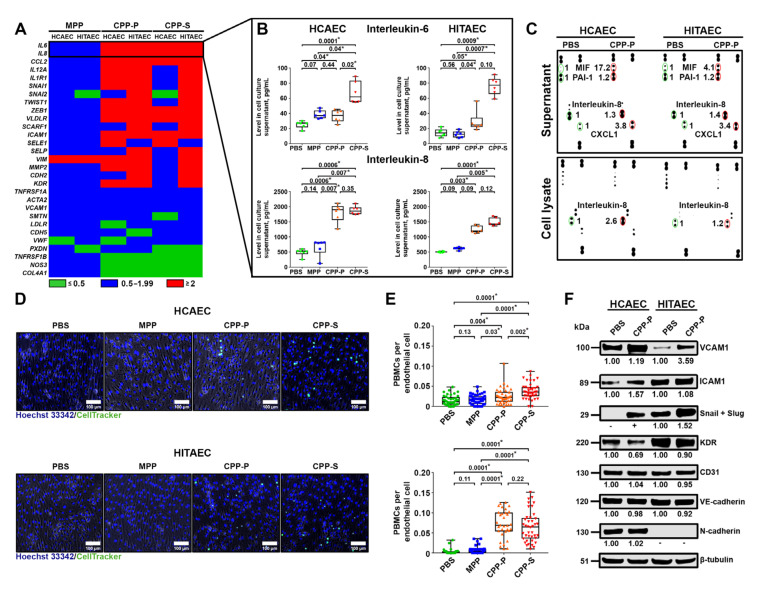
CPPs induce endothelial activation and endothelial-to-mesenchymal transition. (**A**) HCAECs and HITAECs were cultured in the presence of PBS, MPPs, CPP-P, or CPP-S (100 μL of particles per well of a 6-well plate, OD_650_ = 0.08–0.10, for CPP-P and CPP-S: equal to 50 μg calcium or 1.2 × 10^5^ OsteoSense 680EX-positive PKH67-negative events) for 4 h, and total RNA was extracted with the following expression profiling for the indicated genes (n = 3 wells per group). Heat map shows differentially expressed genes (fold change > 2) between groups. (**B**) HCAECs and HITAECs were cultured in the presence of PBS, MPPs, CPP-P, or CPP-S (100 μL of particles per well of a 6-well plate, OD_650_ = 0.08–0.10, for CPP-P and CPP-S: equal to 50 μg calcium or 1.2 × 10^5^ OsteoSense 680EX-positive PKH67-negative events) for 4 h. Conditioned media were profiled for interleukin-6 and -8 using an enzyme-linked immunosorbent assay (n = 6 wells per group). Each dot represents one well of the culture plate. Whiskers indicate the range, box bounds indicate the 25th–75th percentiles, and centre lines indicate the median. *p*-values are provided above the boxes (statistically significant differences are marked by asterisks), Kruskal–Wallis test with post hoc false discovery rate correction by the two-stage linear step-up procedure of Benjamini, Krieger, and Yekutieli. (**C**) Conditioned media and cell lysate from HCAECs and HITAECs from the experiment in (**B**) were profiled using a cytokine array. Note the increase in interleukin-8 in both the supernatant and cell lysate as well as the higher contents of macrophage migration inhibitory factor (MIF) and chemokine (C-X-C motif) ligand 1 (CXCL1) in the supernatant. Numbers indicate densitometry relative to the PBS group. (**D**) HCAECs and HITAECs were cultured in the flow system for 24 h with subsequent treatment with PBS, MPPs, CPP-P, or CPP-S (500 μL of particles per flow system unit, OD_650_ = 0.08–0.10, for CPP-P and CPP-S: equal to 250 μg calcium or 0.6 × 10^6^ OsteoSense 680EX-positive PKH67-negative events) for 4 h and incubation with CellTracker Green CMFDA labelled peripheral blood mononuclear cells (PBMCs) for 1 h. Representative images. Note the considerable adhesion of PBMCs to CPP-P and CPP-S-treated cells. (**E**) Semi-quantitative image analysis of the experiment in (**D**). Each dot represents one image (n = 40 images per group). Whiskers indicate the 25th–75th percentiles, and centre lines indicate the median. *p*-values are provided above boxes (statistically significant differences are marked by asterisks), Kruskal–Wallis test with post hoc false discovery rate correction by the two-stage linear step-up procedure of Benjamini, Krieger, and Yekutieli. (**F**) HCAECs and HITAECs were treated with PBS or CPP-P (1000 μL of particles per T-75 flask, OD_650_ = 0.08–0.10, equal to 500 μg calcium or 1.2 × 10^6^ OsteoSense 680EX-positive PKH67-negative events) for 4 h with further immunoblotting for proteins reflective of endothelial activation and endothelial-to-mesenchymal transition. Note increases in VCAM1 and ICAM1, cell adhesion molecules mediating leukocyte attachment, and Snail and Slug, the key transcription factors regulating endothelial-to-mesenchymal transition. β-tubulin was used as the loading control. Numbers indicate densitometry relative to the PBS group. HCAECs—human coronary artery endothelial cells, HITAECs—human internal thoracic artery endothelial cells, PBS—phosphate-buffered saline, MPPs—magnesiprotein particles, CPP-P—primary calciprotein particles, CPP-S—secondary calciprotein particles, OD_650_—optical density at 650 nm wavelength, CMFDA—5-chloromethylfluorescein diacetate, VCAM1—vascular cell adhesion molecule 1, ICAM1—intercellular cell adhesion molecule 1, KDR—kinase insert domain receptor, VE-cadherin—vascular endothelial cadherin, and N-cadherin—neural cadherin.

In attempts to identify the molecular consequences of CPP internalisation by ECs, we profiled genes engaged in the development of endothelial dysfunction after 4 h of exposure to CPP-P or CPP-S (Figure 8A). Among the differentially expressed genes were those encoding pro-inflammatory cytokines (*IL6*, *IL8*, and *CCL2*), endothelial-to-mesenchymal transition transcription factors (*SNAI1*, *SNAI2*, *TWIST1*, and *ZEB1*), scavenger receptors (*VLDLR* and *SCARF1*), and cell adhesion molecules (*ICAM1*, *SELE*, and *SELP*) (Figure 8A). In agreement with these data, measurement of interleukin (IL)-6 and IL-8 in the cell culture supernatant confirmed their enhanced release by ECs treated with CPP-P or CPP-S but not MPPs (Figure 8B). Among the other increasingly secreted cytokines were macrophage migration inhibitory factor (MIF) and chemokine (C-X-C motif) ligand 1 (CXCL1) (Figure 8C). IL-8 was also found in higher amounts in the lysate of CPP-treated ECs, suggestive of its constant upregulation upon CPP internalisation (Figure 8C). In addition to increasing cytokine release, the addition of CPP-P or CPP-S promoted leukocyte adhesion to ECs in a flow system (Figure 8D,E). Collectively, these findings indicated an endothelial activation that may be associated with a CPP-driven, lysosome-dependent cell death pathway [30,31]. Consistent with the gene expression analysis, cell adhesion molecules VCAM1 and ICAM1 as well as endothelial-to-mesenchymal transition transcription factors Snail and Slug were also overexpressed at the protein level after CPP addition to ECs (Figure 8F).

## 3. Discussion

Elevated levels of serum calcium and phosphate are associated with cardiovascular events and mortality in the general population [1,2,3,4] and subjects with pre-dialysis CKD [32,33,34] or ESRD [35]. However, hyperphosphatemia accounts for medial arterial calcification [6], which is also common in patients with CKD and ESRD [36], but does not provoke atherosclerosis [6] or intimal calcification [37]. The mechanism of accelerated coronary artery disease or cerebrovascular disease development in patients with altered mineral homeostasis was previously unclear. Therefore, a need remained to identify mineral-related factors capable of triggering endothelial dysfunction and adventitial/perivascular inflammation, the two major drivers of atherosclerosis [11,26,38].

Insights into the molecular basis of extraskeletal calcification inhibition pinpointed the central role of fetuin-A in the maintenance of mineral homeostasis [7,8]. Interactions of fetuin-A with calcium and phosphate result in crystalline nanosized CPPs, which represent a vehicle for the clearance of excessive serum calcium and phosphate [21]. In vitro and in vivo experiments demonstrated that CPPs are unable to cause cardiovascular calcification per se, attesting their protective significance [10]. Nevertheless, CPPs exerted significant cytotoxic effects on ECs [10]. These findings imply an ambiguous role of CPPs in vascular physiology. We hypothesised that while CPPs guard blood vessels from medial calcification, which rapidly causes mechanical incompetence of the arteries, these mineral complexes may also have pathogenic effects ultimately disrupting vascular homeostasis by promoting endothelial dysfunction and adventitial or perivascular inflammation.

We first sought to explore the clinical relevance of CPP formation in a CVD setting. To this end, we applied a simple assay including a single measurement of optical density increase after 24 h of serum incubation in cell culture conditions following the addition of calcium salts and phosphates, thereby evaluating CPP generation propensity. Patients with coronary artery disease or cerebrovascular disease were characterised by considerably higher CPP yield than healthy blood donors because of increased ionised calcium and reduced albumin, a protein playing a pivotal role in preventing extraskeletal mineralisation by Ca^2+^ binding. Notably, the CVD risk pattern in relation to CPP, ionised calcium, and albumin levels was reminiscent of that observed for serum calcium and phosphate in population studies [1,2,3,4], whereas phosphate and fetuin-A concentrations did not affect cardiovascular risk in our investigation. Taken together, these findings suggest insufficient Ca^2+^-binding capacity as a leading mechanism disturbing mineral homeostasis in patients with CVD and promoting CPP formation. As elevated CPP production was also associated with unstable coronary plaque phenotype, we propose that our approach can be implemented in clinical trials both for CVD risk assessment and for higher-accuracy prognostication in patients with coronary artery disease. Intriguingly, serum CPP generation propensity was significantly higher in patients with cerebrovascular disease in comparison with those suffering from coronary artery disease (including those with myocardial infarction). The reason for this finding may include the greater reduction in total protein and albumin, probably because of the lower eGFR (average 74.00 in cerebrovascular disease patients vs. 86.00 in coronary artery disease patients), though these values still did not reach those defining clinically significant CKD. Although reduced eGFR might be partially responsible for the higher CPP generation propensity in patients with cerebrovascular disease or myocardial infarction, the restriction of patient and control cohorts to the subjects having an eGFR >90 mL/min/1.73 m^2^ did not change the results, as cardiovascular disease patient cohorts still demonstrated higher CPP generation propensity, increased serum ionised calcium, and reduced total protein and albumin levels. Hence, we found no conclusive evidence that eGFR considerably influences CPP formation in the serum. Nevertheless, these findings require replication in subsequent epidemiological studies, ideally in a comprehensive multicentre research trial aimed at the evaluation of mineral homeostasis parameters in patients with cardiovascular disease and healthy volunteers.

To exclude the possible confounding impacts of cardiovascular risk factors, we intravenously injected CPPs to normolipidemic and normotensive Wistar rats employing various protocols, some of which included artificial vascular injury. Regardless of CPP administration regimen or endothelial integrity, these mineral particles provoked an intimal hyperplasia and adventitial/perivascular inflammation, reflected by increased VV and MCs. These results provide a proof of concept that aggregation of calcium and phosphate into the CPPs disrupts vascular homeostasis, although the details of CPP fate starting from their formation in the blood until their internalisation by ECs are yet to be elucidated.

Subsequent experiments on human arterial endothelial cell cultures confirmed the pronounced endothelial toxicity of CPPs (but not MPPs) upon their dissolution in lysosomes, suggesting a mechanism for the deleterious consequences of CPP formation. In agreement with recently acknowledged molecularly oriented definitions of cell death subroutines [17], we defined the CPP-induced mode of cellular demise as lysosome-dependent cell death, demarcated by primary lysosomal membrane permeabilisation and mediated via executioner caspases. As lysosomal inhibitor bafilomycin A1 abrogated cell death in CPP-treated cells while not endowing MPPs with cytotoxicity, we inferred and showed that the translocation of Ca^2+^ ions from the lysosomes to the cytosol triggered mitochondrial outer membrane permeabilisation, evidenced by XIAP downregulation and followed by cleavage of caspase-3, further implementing the regulated cell death. Our prior results denoted the concomitant cleavage of caspase-9 in support of these observations [10]. These findings are concordant with those obtained earlier for vascular smooth muscle cells [39] but contradict the previous assumptions that the dissolution of CPPs provokes lysosomal rupture by creating an osmotic pressure gradient between the lysosomes and cytosol [40] (since the dissolution of MPPs would also simulate this scenario, yet these particles lack cytotoxicity). Another mechanism of CPP-related cytotoxicity may be the generation of reactive oxygen species [24,41]; however, we did not document conclusive signs of this phenomenon.

The augmented release of proinflammatory cytokines (i.e., IL-6, IL-8, MIF, and CXCL1) enhanced leukocyte adhesion and increased the expression of transcription factors Snail and Slug, suggesting endothelial activation and endothelial-to-mesenchymal transition as prominent molecular consequences upon CPP internalisation by ECs. This corresponds to earlier data obtained on ECs [10], vascular smooth muscle cells [42], and macrophages [24], collectively suggesting chronic vascular inflammation as a major mechanism mediating the pathogenic effects of CPPs on blood vessels. Yet, vascular cell populations showed a differential response to CPPs, as ECs demonstrated an augmented production of IL-6 and IL-8, while perivascular adipocytes ceased IL-8 secretion upon CPP treatment. The orchestration of vascular inflammation by CPPs (e.g., the paracrine effects of CPP-exposed ECs, vascular smooth muscle cells, adventitial macrophages, and perivascular adipocytes) and its molecular mechanisms in vivo are yet to be elucidated. Although CPPs mount a pro-inflammatory response in ECs [10], vascular smooth muscle cells [42], and macrophages [19,24,41], these effects may be cell- and context-specific and require further investigation in co-culture or conditioned medium experiments.

In contrast to magnesium phosphate hydrate particles containing a negligible amount of calcium (MPPs), both amorphous and spherical aggregates of calcium, phosphate, and serum proteins (CPP-P) and crystalline and spindle-shaped calcium phosphate protein-coated particles (CPP-S) caused pathogenic effects on arteries upon intravenous administration to normolipidemic/normotensive animals and after the addition to EC cultures. The pathogenicity of CPPs for ECs regardless of their ripening suggests that these particles have detrimental effects immediately upon their formation, thereby emphasising the clinical relevance of excessive serum’s propensity to form CPPs.

The limitations of our study include:

- We did not apply the T_50_ test, which reflects the rate of transformation of CPP-P to CPP-S in the human serum, as we think the total propensity of serum to form CPPs (measured by OD_650_ increment) is a suitable and pathophysiologically relevant alternative that might be less sophisticated for routine clinical use. The T_50_ test has its advantages (e.g., validation of multiple patient cohorts), yet the mineral stress test (OD_650_ increase) applied in this study is not contradictory and represents another variation in serum calcification propensity measurement.
- The concentration of +2 mmol/L CaCl_2_ and Na_2_HPO_4_ added is supraphysiological and such excessive Ca/P concentrations are rarely encountered, except in patients with severe osteopenia/osteoporosis and hyperparathyroidism who frequently show hypercalcemia and those with ESRD who generally suffer from hyperphosphatemia. Yet, this Ca/P increase represents a mineral stress test for the measurement of serum’s propensity to form CPPs; lower amounts of added CaCl_2_ and Na_2_HPO_4_ concentrations inconsistently triggered CPP generation. Increased CPP generation propensity (higher OD_650_ increase values) was associated with higher ionised calcium (Ca^2+^) and lower albumin, thereby reflecting disturbed mineral homeostasis in cohorts of patients with coronary artery disease and cerebrovascular disease compared with healthy blood donors.
- In our study, we did not perform animal section immunostainings as these sections were formalin-fixed for significantly longer than 24 h and therefore were unsuitable for IHC-P or IHC-Fr applications, although electron microscopy analysis well-distinguishes blood microvessels from the background by the combination of clearly visible vessel lumen, endothelial layer, and (optionally) red blood cells within the lumen. In addition, macrophages also have morphological patterns distinct from other adventitial cell populations (e.g., fibroblasts and lymphocytes), in particular when assembled into clusters, which is not characteristic for fibroblasts. IHC stainings of the aortic sections from another experiment showed positive staining of MCs for F4/80 and myeloperoxidase but not for T lymphocyte CD3 marker (Figure 5E, insets).
- We did not study CPP’s effects on vascular smooth muscle cells. However, we stained aortas from both animal models with alizarin red S and found no signs of vascular calcification, suggesting that the osteogenic differentiation of vascular smooth muscle cells is not among the major consequences of CPP administration in vivo. In a balloon injury model, endothelial denudation and mechanical vascular injury collectively led to intimal hyperplasia because of damaged internal elastic lamina and the possible proliferative activation of vascular smooth muscle cells. We suggest that exposure of balloon-injured blood vessel to CPPs might also contribute to the contractile-to-synthetic phenotypic switch of vascular smooth muscle cells, although we could not confirm this because the sections could not be used for immunohistochemistry. Whether this scenario occurs in intact blood vessels is currently unclear, whereas endothelial dysfunction might result in pathological paracrine signalling to adjacent vascular smooth muscle cells.

**Figure 9:**
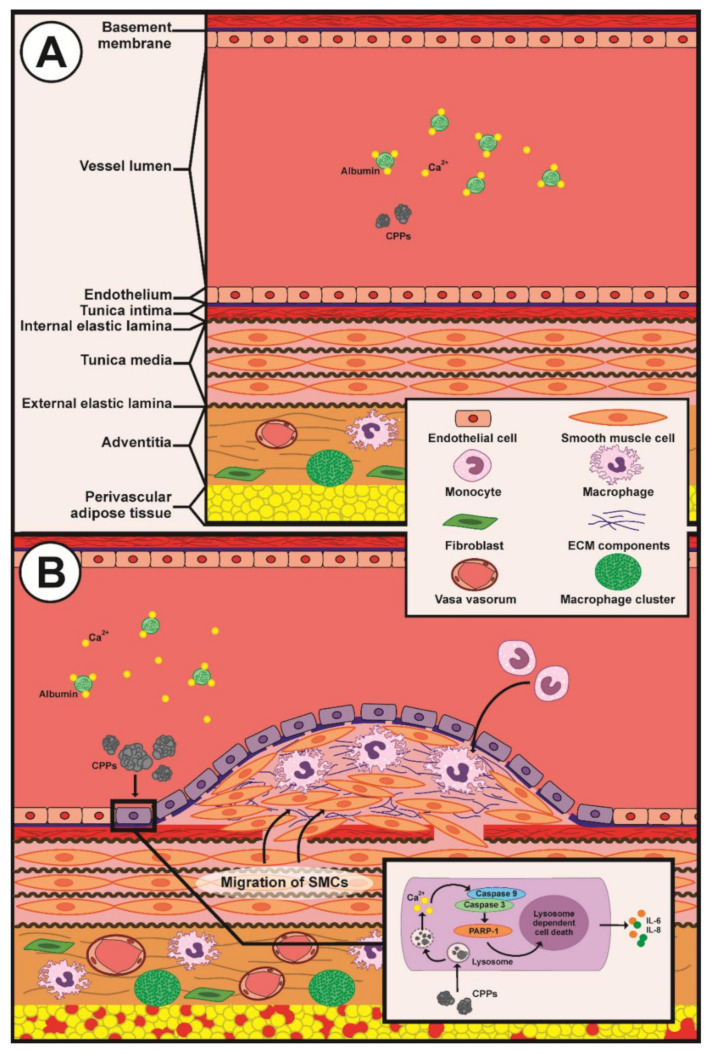
An illustration depicting the putative mechanism of CPP-induced vascular injury in patients with cardiovascular disease. (**A**) Physiological scenario in healthy individuals; (**B**) pathological scenario in patients with cerebrovascular disease or coronary artery disease and reduced Ca^2+^-binding capacity. Supersaturation with Ca^2+^ ions due to reduced serum Ca^2+^-binding capacity leading to CPP aggregation in the blood. CPPs are internalised by the endothelial cells causing their lysosome-dependent death, endothelial activation, and endothelial-to-mesenchymal transition. This provokes leukocyte adhesion and the excessive release of proinflammatory cytokines interleukin-6 and -8, collectively promoting chronic inflammation and neointima formation. Furthermore, CPPs cause adventitial and perivascular inflammation reflected by the expansion of the vasa vasorum and macrophage clusters, which also contributes to intimal hyperplasia and disrupted vascular homeostasis.

To summarise (Figure 9), we here found that patients with CVD are prone to the formation of CPPs as a result of disturbed mineral homeostasis (in particular to reduced Ca^2+^-binding capacity). In other words, decreased albumin (the most abundant Ca^2+^-binding protein) leads to the reciprocal increase in ionised calcium (Ca^2+^), which provides a chemical substrate for the elevated CPP generation. We for the first time showed that CPPs are able to cause intimal hyperplasia due to endothelial dysfunction and adventitial/perivascular inflammation, potentially through the increased leukocyte adhesion and excessive release of pro-inflammatory cytokines by ECs. Hence, we propose CPPs as a mechanistic link explaining the increased incidence of CVD in individuals with altered mineral homeostasis. We suggest disturbed mineral homeostasis (in particular reduced albumin, increased ionised calcium, and elevated serum CPP formation propensity) as an additional risk factor of cardiovascular disease, although its contribution might be less significant in comparison with other major established cardiovascular risk factors (i.e., dyslipidaemia, arterial hypertension, overweight/obesity, and carbohydrate metabolism disorders), in concert with the epidemiological studies that showed an association between increased calcium, decreased albumin, and major adverse cardiovascular events [1,2,3,4]. The pathophysiological importance of excessive CPP formation in experimental cardiovascular disease settings and relevant clinical scenarios is underscored by the association of elevated CPP formation propensity with coronary artery disease and cerebrovascular disease (especially myocardial infarction and ischaemic stroke), the development of intimal hyperplasia in normolipidemic/normotensive rats upon regular intravenous administration of CPPs, the aggravation of intimal hyperplasia in balloon-injured aortas after CPP injections, and the pronounced endothelial dysfunction in primary arterial EC cultures treated with CPPs.

From a translational perspective, CPPs may be targeted with Ca^2+^ chelators, which demonstrated certain efficiency in the prevention of cardiovascular events after index myocardial infarction [43], especially in patients with a concomitant diabetes mellitus [44] and peripheral artery disease [45]. Another promising therapeutic option is Mg^2+^ supplementation, which delays the maturation of primary-to-secondary CPPs in a dose-dependent manner [46], reduces CPP load and abates inflammation in haemodialysed patients [47], and inhibits vascular calcification in uremic rats [48]. Alternatively, 4,6-di-O-(methoxy-diethyleneglycol)-myo-inositol-1,2,3,5-tetrakis(phosphate), an inositol phosphate analogue, displayed similar effects [49]. A recent study showed that replacement of calcium carbonate with lanthanum carbonate as a phosphate binder lowers serum CPP levels in patients with ESRD [50], in addition to attenuating aortic calcification [51]. Collectively, our results provide a rationale for the development and testing of the aforementioned and novel modalities of anti-CPP therapy potentially beneficial for patients with disturbed mineral homeostasis.

## 4. Materials and Methods

### 4.1. Evaluation of Serum Propensity for Calciprotein Particle (CPP) Formation

This study was approved by the local ethical committee of the Research Institute for Complex Issues of Cardiovascular Diseases (Kemerovo, Russia, protocol numbers 20160404 and 20180803, dates of approval: 4 April 2016 and 3 August 2018, respectively), and a written informed consent was provided by all study participants after receiving a full explanation of the study. The investigation was carried out in accordance with the Good Clinical Practices and the Declaration of Helsinki. The criteria for inclusion were: (1) performance of carotid endarterectomy due to brain ischemia or ischemic stroke or coronary artery bypass graft surgery because of stable angina or hospitalisation due to myocardial infarction or participance in the Prospective Urban Rural Epidemiology Study in combination with the absence of symptomatic carotid or coronary atherosclerosis; (2) a signed written informed consent to be enrolled. One criterion of exclusion was incomplete investigation regardless of the reason; in this case, we enrolled another subject with similar age, sex, and clinicopathological features who met the inclusion criteria. In total, we consecutively enrolled 88 healthy volunteers, 44 patients with brain ischemia who required carotid endarterectomy, 44 patients with ischemic stroke, 44 patients with stable angina who required coronary artery bypass graft surgery, and 44 patients with myocardial infarction.

Cerebrovascular disease (brain ischemia and ischemic stroke), stable angina, and myocardial infarction, as well as comorbid conditions (arterial hypertension, chronic heart failure, chronic obstructive pulmonary disease, asthma, chronic kidney disease, diabetes mellitus, overweight, and obesity) were diagnosed and treated according to the respective guidelines of European Society of Cardiology, Global Initiative for Chronic Obstructive Lung Disease, Global Initiative for Asthma, Kidney Disease: Improving Global Outcomes, American Diabetes Association, and European Association for the Study of Obesity. eGFR was calculated according to the Chronic Kidney Disease Epidemiology Collaboration (CKD-EPI) equation. The left ventricular ejection fraction was evaluated by means of echocardiography (Sonos 2500 Diagnostic Ultrasound System, Hewlett Packard, Palo Alto, CA, USA). The number of affected coronary arteries in patients with myocardial infarction was defined during coronary angiography (Innova 3100 Cardiac Angiography System, General Electric Healthcare, Chicago, IL, USA), whereas extracranial artery stenosis in those with cerebrovascular disease was assessed using colour duplex screening (Vivid 7 Dimension Ultrasound System, General Electric Healthcare, Chicago, IL, USA). Data on age, sex, smoking status, and pharmacological anamnesis were collected at the time of admission. The detailed characteristics of the study sample are presented in Table S2.

To determine the optimal amount of added CaCl_2_ and Na_2_HPO_4_ for the assay, the serum of healthy (i.e., asymptomatic) volunteers (n = 49) was supersaturated with calcium and phosphate (0.5, 1, 2, 3, 4, or 5 mmol/L) by adding equal concentrations of CaCl_2_ (21115, Sigma-Aldrich, St. Louis, MO, USA) and Na_2_HPO_4_ (94046, Sigma-Aldrich, St. Louis, MO, USA). Upon 24 h incubation in cell culture conditions (37 °C, 5% CO_2_, and high humidity), we monitored the change in optical density at a wavelength of 650 nm (OD_650_, Multiskan Sky, Thermo Fisher Scientific, Waltham, MA, USA) s compared to the same serum without calcium/phosphate supplementation. To evaluate CPP formation over time, equimolar (2 mmol/L) concentrations of CaCl_2_ and Na_2_HPO_4_ were added to the serum of healthy individuals (n = 42) with the subsequent time-lapse (1 h time frame) measurement of OD_650_ during 24 h and calculation of the: (1) average one-half maximal transition time (T_50_) reflecting the average time required for reaching the one-half of the maximal OD_650_ increase across the samples; (2) one-half maximal OD_650_ increase reflecting the average OD_650_ value equal to the half of the maximal OD_650_ increase across the samples. To characterise serum-generated CPPs, we diluted the supersaturated and 24 h incubated serum 4-fold to reduce its viscosity, centrifuged it at 200,000× *g* for 2 h (Optima MAX-XP, Beckman Coulter, Brea, CA, USA), and lysed the sediment in RIPA buffer (89901, Thermo Fisher Scientific, Waltham, MA, USA) for 30 min to lyse the extracellular vesicles. Then, the lysate was centrifuged again at 200,000× *g* for 1 h, and the pellet was washed in sterile-filtered double-distilled water with the following centrifugation at 200,000× *g* for 1 h. The serum-generated CPPs were characterised by means of scanning electron microscopy (S-3400N, Hitachi, Tokyo, Japan) and elemental analysis (energy-dispersive X-ray spectroscopy, XFlash 4010, Bruker, Billerica, MA, USA) upon the resuspension of washed CPP pellet in sterile-filtered double distilled water and pipetting the resuspended CPPs on a double-sided adhesive conductive carbon tape (16084-7, Ted Pella, Redding, CA, USA).

Equimolar (2 mmol/L) concentrations of CaCl_2_ and Na_2_HPO_4_ were added to the serum of healthy volunteers (n = 88) or patients with brain ischemia (n = 44), ischemic stroke (n = 44), stable angina (n = 44), or myocardial infarction (n = 44) in 96-well plates (100 μL per well). Upon 24 h incubation in cell culture conditions, we monitored the change in OD_650_ compared to the same serum without calcium/phosphate supplementation. Serum concentrations of ionised calcium, phosphate, total protein, and albumin were measured using an automated biochemical analyser (Konelab 60i, Thermo Fisher Scientific, Waltham, MA, USA), whereas fetuin-A level was determined by enzyme-linked immunosorbent assay (RD191037100, BioVendor, Heidelberg, Germany) according to the manufacturer’s protocol.

### 4.2. Artificial Synthesis and Quantification of CPPs

To synthesise primary (CPP-P) and secondary (CPP-S) CPPs, stock solutions of CaCl_2_ and Na_2_HPO_4_ were diluted to equal concentrations of 3 (CPP-P) or 7.5 (CPP-S) mmol/L in Dulbecco’s modified Eagle’s medium (DMEM, 31330038, Thermo Fisher Scientific, Waltham, MA, USA) supplemented with 10% (CPP-P) or 1% foetal bovine serum (CPP-S). For the synthesis of magnesiprotein particles (MPPs), stock solutions of MgCl_2_ (97062-848, VWR, Radnor, PA, USA) and Na_2_HPO_4_ were diluted to equal concentrations of 20 mmol/L in DMEM supplemented with 10% foetal bovine serum (FBS, 10270106, Thermo Fisher Scientific, Waltham, MA, USA). The reagents were added into DMEM in the following order: (1) FBS; (2) CaCl_2_ or MgCl_2_; (3) Na_2_HPO_4_, with a vortexing between the added reagents. Following 24 h incubation in cell culture conditions, the medium was centrifuged at 200,000 *g* for 1 h (Optima MAX-XP, Beckman Coulter, Brea, CA, USA), and the particle sediment was resuspended in double-distilled water for the microscopy and chemical analysis, phosphate-buffered saline (PBS) for cell culture experiments, or 0.9% NaCl for animal studies. Quantification of CPPs and MPPs was performed using three approaches: (1) OD_650_ measurement of MPP/CPP suspension; (2) determination of Ca^2+^ amount per microlitre of MPP/CPP suspension by means of the respective colorimetric kit (ab102505, Abcam, Cambridge, U.K.) at an optical density of 575 nm; (3) quantitation of OsteoSense 680EX-positive PKH67-negative events per microlitre of MPP/CPP suspension in strict accordance with a recent fluorescent probe-based flow cytometry assay [ Briefly, 15 μL of the CPP suspension was added to 75 μL sterile-filtered Tris-buffered saline (pH 7.4); then, 67 μL of this mix was blended with 83 μL fluorescent-labelled bisphosphonate OsteoSense 680EX (1:75 dilution, NEV10020EX, PerkinElmer, Waltham, MA, USA) and incubated in the dark for 50 min at 4 °C, with the subsequent addition of 8.3 μL lipophilic dye PKH67 (1:100 dilution, MIDI67-1KT, Sigma-Aldrich, St. Louis, MO, USA) and further incubation in the dark for another 10 min at 4 °C before sample acquisition (CytoFLEX, Beckman Coulter, Brea, CA, USA). In this experimental setup, OsteoSense 680EX bound to CPPs while PKH67 discriminated CPPs from similar-sized extracellular vesicles; therefore, CPPs were defined as OsteoSense 680EX-positive PKH67-negative events. Generally, OD_650_ values of 0.08–0.10 (≈0.5 μg/μL calcium and ≈1.2 × 10^3^ OsteoSense 680EX-positive PKH67-negative events/μL for both CPP-P and CPP-S) were considered as proper for the experimentation as this is the minimum reliable threshold of the particle density in the solution. MPPs were almost devoid of calcium (≈0.03 μg/μL calcium and <10 OsteoSense 680EX-positive PKH67-negative events/μL). The concept was to apply as low a dose of CPPs as possible.

### 4.3. Electron and Atomic Force Microscopy

MPPs, CPP-P, and CPP-S were visualised using scanning electron microscopy (SEM), transmission electron microscopy (TEM), and atomic force microscopy (AFM). For SEM, we pipetted a few drops of the particle suspension on a glass microscope slide, dried the slides at room temperature overnight, mounted the slides on a double-sided adhesive conductive carbon tape (16084-7, Ted Pella, Redding, CA, USA), sputter-coated with gold and palladium (EM ACE200, Leica Microsystems, Wetzlar, Germany), and finally performed the SEM (SU8220, Hitachi, Tokyo, Japan). For TEM, we pipetted a few drops of the particle suspension on a carbon-coated copper grid (3520C-FA, Structure Probe, Inc., West Chester, PA, USA), stained the sample with 2% uranyl acetate (22400-2, Electron Microscopy Sciences, Hatfield, PA, USA) for 20 min, and carried out the TEM (JEM-2100, Jeol, Tokyo, Japan). For AFM, we pipetted a few drops of the particle suspension on a mica disc (50-12, Ted Pella, Redding, CA, USA), and conducted the AFM (Cypher, Asylum Research, Santa Barbara, CA, USA).

### 4.4. Measurement of Particle-Size Distribution and Surface Charge

The particle-size distribution curve and surface charge (zeta potential) of MPPs, CPP-P, and CPP-S were assessed by dynamic and electrophoretic light scattering, respectively (Zetasizer Nano ZS, Malvern Instruments, Malvern, U.K.). Before the measurement, samples were incubated at 25 °C for 10 min. All measurements were performed thrice (30 runs per measurement) with the further calculation of the average distribution.

### 4.5. Chemical Profiling

The chemical elements composing MPPs, CPP-P, and CPP-S were determined by energy-dispersive X-ray spectroscopy (EDX), atomic emission spectroscopy (AES), and CHNSO analysis. For EDX, we pipetted a few drops of the particle suspension on a double-sided adhesive conductive carbon tape, dried it for 2 h at 37 °C, and performed elemental analysis (XFlash 4010, Bruker, Billerica, MA, USA). For each sample, we defined three quadrants where particles were clearly observed, and then calculated the average atomic percent for each element. AES (iCAP 6500, Thermo Fisher Scientific, Waltham, MA, USA) was carried out upon dissolution of the particles in HNO_3_ (438073, Sigma-Aldrich, St. Louis, MO, USA) for 1 h at 80 °C. CHNSO analysis was performed by catalytic oxidation of the particles at 1060 °C (Flash 2000, Thermo Fisher Scientific, Waltham, MA, USA).

To determine which functional groups were formed by the particle-associated elements, we applied Fourier transform infrared spectroscopy (FTIR, Vertex 80v, Bruker, Billerica, MA, USA) and Raman spectroscopy (LabRam HR800, Horiba Scientific, Piscataway, NJ, USA). FTIR and Raman spectra were obtained at a resolution of 4 cm^−1^ (FTIR) or 0.222 cm^−1^ (Raman spectroscopy) and at wavelengths ranging from 4000 to 500 cm^−1^ (FTIR) or 100 cm^−1^ (Raman spectroscopy).

The chemical formulas of the compounds that constitute MPPs, CPP-P, and CPP-S were deciphered by X-ray powder diffractometry (XRD, D8 ADVANCE, Bruker, Billerica, MA, USA) with an X-ray copper tube operating at 40 kV. Data were collected over a 2θ angle ranging from 20 to 120° at a speed of 0.02°/s. Diffraction spectra were compared with the database of the Joint Committee on Powder Diffraction and Standards. Particle crystallinity was defined during TEM by an analysis of the selected area diffraction patterns that resulted from the electron beam scattered by the sample lattice.

The protein content of CPPs and MPPs was defined by sodium dodecyl sulphate-polyacrylamide gel electrophoresis (SDS-PAGE) with subsequent silver staining. Equal aliquots (20 μL, OD_650_ = 0.08–0.10, for CPP-P and CPP-S: equal to 10 μg calcium or 2.4 × 10^4^ OsteoSense 680EX-positive PKH67-negative events) of MPPs, CPP-P, or CPP-S suspensions were mixed with NuPAGE LDS sample buffer (NP0007, Thermo Fisher Scientific, Waltham, MA, USA) in a 4:1 ratio and a NuPAGE sample reducing agent (NP0009, Thermo Fisher Scientific, Waltham, MA, USA) at a 10:1 ratio, and then loaded on a 1.5 mm NuPAGE 4–12% Bis-Tris protein gel (NP0335BOX, Thermo Fisher Scientific, Waltham, MA, USA). Precision Plus protein standard (1610374, Bio-Rad, Hercules, CA, USA) was loaded as a molecular weight marker. Proteins were separated by SDS-PAGE at 100 V for 1 h. The gel was stained using a Silver Stain Plus staining kit (1610449, Bio-Rad, Hercules, CA, USA) according to the manufacturer’s protocols. Ethylenediaminetetraacetic acid disodium salt (E4884, Sigma-Aldrich, St. Louis, MO, USA) at a 39 mmol/L concentration was added to stop the reaction. Gels were photographed using an HP Scanjet Enterprise Flow 7500 Flatbed Scanner (Hewlett Packard, Palo Alto, CA, USA).

The extraction of lipids was performed by the Folch method using a conventional protocol. Gas chromatography-mass spectrometry (GC-MS) was performed using an MDN-1 column (nonpolar methylsilicone, 30 m × 0.25 mm, 24259, Sigma-Aldrich, St. Louis, MO, USA) and a GCMS-QP2010 Ultra (Shimadzu, Kyoto, Japan) according to the following parameters: injection volume, 1 μL; injector temperature, 200 °C; split ratio, 1:10; interface temperature, 210 °C; detector temperature, 200 °C; carrier (He) flow rate, 0.8 mL/min; and temperature program, 100 °C for 2 min, 5°/min up to 120 °C, 20°/min up to 260 °C, then 260 °C for 2 min. Mass range was from 1.5 to 1900 *m*/*z*.

### 4.6. Animal Models

Male Wistar rats weighing 250–300 g, 12–14 weeks of age, provided by the Research Institute for Complex Issue of Cardiovascular Diseases Core Facility, were used for all animal experiments (n = 130). Animals were allocated to polypropylene cages (5 rats per cage) lined with wood chips and had access to the water and food (rat chow) ad libitum. Throughout the duration of the experiment, the standard conditions of temperature (24 ± 1 °C), relative humidity (55 ± 10%), and 12 h light/dark cycles were carefully maintained, and the health status of all rats was monitored daily. No randomisation was performed to allocate animals to experimental groups or cages. There were no specific inclusion or exclusion criteria. Experiments were performed in a blinded fashion. All procedures were approved by the Local Ethical Committee of the Research Institute for Complex Issues of Cardiovascular Diseases (Kemerovo, Russia, protocol number 20160404, date of approval: 4 April 2016). All procedures conformed to the guidelines of Directive 2010/63/EU of the European Parliament on the protection of animals used for scientific purposes and to the NIH Guide for the Care and Use of Laboratory Animals.

To assess whether CPPs are able to aggravate pre-existing endothelial injury, we applied an experimental model of rat aorta angioplasty with a coronary angioplasty balloon catheter. After the induction of anaesthesia with 3% isoflurane (Aerrane), all animals received inhalation anaesthesia with 1.5% isoflurane throughout surgery. Briefly, the aorta was punctured in the proximal direction with a 21-gauge needle; a DIOR 2.0 × 15 mm balloon catheter with a 0.014 inch guidewire was then inserted into the aortic lumen, and an angioplasty was finally carried out with inflation pressure of 5 atm for 30 sec. Immediately after the surgery, a suspension of MPPs, CPP-P, or CPP-S (900 μL, OD_650_ = 0.08–0.10, for CPP-P and CPP-S: equal to 450 μg calcium or 1.08 × 10^6^ OsteoSense 680EX-positive PKH67-negative events) or equal volume of 0.9% NaCl (n = 10 rats per group, 40 rats in total) was injected into the tail vein.

Five weeks postoperation, all rats were euthanised by an intraperitoneal injection of a sodium pentobarbital (100 mg/kg body weight). At the site of injury, the aorta was excised and fixed in two changes of 10% neutral phosphate-buffered formalin for 24 h at 4 °C, dehydrated in ascending ethanol series (70%, 80%, and 95%; 1 h each) and isopropanol (1 h), impregnated and embedded into paraffin (3 changes, 1 h each, Paraplast REGULAR, 39601006, Leica Biosystems, Wetzlar, Germany), cooled at 4 °C overnight, frozen at −20 °C, and cut (5 μm sections) on a microtome (Microm HM 325, Thermo Fisher Scientific, Waltham, MA, USA). To ensure proper histological examination, we prepared 12 sections, evenly distributed across the entire aortic segment, per slide. Sections were then H&E- and alizarin red S stained (ab245880 and ab146374, Abcam, Cambridge, U.K.) according to the manufacturer’s protocols for general examination. Sections were evaluated by light microscopy (AxioImager.A1, Carl Zeiss, Stuttgart, Germany) in a blinded fashion for the extent of intimal hyperplasia or adventitial/perivascular inflammation.

Neointimal area as well as total number and area of macrophage clusters were evaluated using ImageJ software (National Institutes of Health, Bethesda, MD, USA). Intimal hyperplasia was defined as neointimal area ≥5000 μm^2^, as these values indicate a clearly visible neointimal lesion. Macrophage phenotype in such clusters was verified by immunohistochemistry (Novolink Max Polymer Detection System, RE7280-K, Leica, Wetzlar, Germany) after antigen retrieval (ab93678, Abcam, Cambridge, UK) according to the manufacturers’ protocols using the antibodies to a macrophage marker F4/80 (ab100790, Abcam, Cambridge, U.K.; 1:200 dilution), macrophage activity-reflecting enzyme myeloperoxidase (ab208670, Abcam, Cambridge, U.K.; 1:200), pan-leukocyte marker CD45 (ab10558, Abcam, Cambridge, U.K.; 1:200), and T cell marker CD3 (ab16669, Abcam, Cambridge, U.K.; 1:200). Haematoxylin was used as the counterstain.

Alternatively, to test the ability of CPPs to cause endothelial injury per se, we performed consecutive tail vein injections of MPPs, CPP-P, or CPP-S (900 μL of particles per injection, OD_650_ = 0.08–0.10, for CPP-P and CPP-S: equal to 450 μg calcium or 1.08 × 10^6^ OsteoSense 680EX-positive PKH67-negative events) or an equal volume of 0.9% NaCl (once or thrice per week during 5 weeks, n = 10 rats per group, 70 rats in total) without any surgical intervention. Five weeks following the start of the injections, all rats were euthanised, and aortic arches and descending aortas (i.e., aortic segments with a turbulent and laminar flow, respectively) were excised and treated as described above. Sections were then examined for the presence of intimal hyperplasia.

As the proper examination of the vasa vasorum requires electron microscopy, which is incompatible with routine histological examination, for the comprehensive investigation of adventitial/perivascular inflammation, we applied another animal model in which rats underwent balloon angioplasty followed by daily tail vein injections of MPPs, CPP-P, or CPP-S (900 μL of particles per injection, OD_650_ = 0.08–0.10, for CPP-P and CPP-S: equal to 450 μg calcium or 1.08 × 10^6^ OsteoSense 680EX-positive PKH67-negative events) or an equal volume of 0.9% NaCl for 5 days (n = 5 rats per group, 20 rats in total). Rats were euthanised 5 weeks postoperation as described above, with subsequent excision of the injured and intact aortic segments. Vessels were then fixed in two changes of 10% neutral phosphate-buffered formalin for 24 h at 4 °C, postfixed in 1% osmium tetroxide (OsO_4_, 19110, Electron Microscopy Sciences, Hatfield, PA, USA) for 24 h, stained in 2% osmium tetroxide for 48 h, dehydrated in ascending ethanol series (50%, 60%, 70%, 80% and 95%, 15 min each), stained in 2% alcoholic uranyl acetate for 5 h, dehydrated in isopropanol (5 h) and acetone (1 h), impregnated with an acetone:epoxy resin (Epon, 14120, Electron Microscopy Sciences, Hatfield, PA, USA) mixture (1:1) for 6 h and with epoxy resin for 24 h, and were finally embedded into fresh epoxy resin at 60 °C. Samples were then ground, polished (TegraPol-11, Struers, Copenhagen, Denmark), and counterstained with Reynolds’s lead citrate (17810, Electron Microscopy Sciences, Hatfield, PA, USA) for 7 min. After a washing in double-distilled water, samples were sputter-coated (10 nm thickness) with carbon (EM ACE200, Leica Biosystems, Wetzlar, Germany) and visualised by means of backscattered scanning electron microscopy at a 10 kV voltage (S-3400N, Hitachi, Tokyo, Japan). The total number and area of VV and MCs in the examined vessels, further normalised by the area of adventitia and perivascular adipose tissue to calculate VV and MC density, were evaluated using ImageJ software (National Institutes of Health, Bethesda, MD, USA). VV was defined by the combination of clearly visible vessel lumen, endothelial layer, and (optionally) red blood cells within the lumen.

### 4.7. Cell Culture

Primary cultures of human coronary artery endothelial cells (HCAECs, 300K-05a, Cell Applications, San Diego, CA, USA) and human internal thoracic artery endothelial cells (HITAECs, 308K-05a, Cell Applications, San Diego, CA, USA) were cultured according to the manufacturer’s protocols. For the extraction of primary perivascular adipocytes, adipose tissue samples (2 mm^3^) obtained from the coronary artery and internal mammary artery anastomosis during the coronary artery bypass graft surgery (the patient provided a written informed consent for the procedure after receiving a full explanation of the study, Local Ethical Committee of the Research Institute for Complex Issues of Cardiovascular Diseases (Kemerovo, Russia) protocol number 20190704, date of approval: 4 July 2019) were incubated in a collagenase solution (0.5 mg/mL, 17100017, Thermo Fisher Scientific, Waltham, MA, USA) containing 200 nmol/L adenosine (1160CBC, Merck Millipore, Burlington, MA, USA) in a water bath at 37 °C for 30 min. The floated fraction of the digested perivascular adipose tissue was then transferred into M199 culture medium (12340030, Thermo Fisher Scientific, Waltham, MA, USA) supplemented with glucose (5 mmol/L) and 10% FBS and filtered through a 100 μm cell strainer (21008-950, VWR, Radnor, PA, USA). The flow-through containing perivascular adipocytes was further centrifuged for 2 min at 200× *g*, resuspended in 1 mL culture medium, and finally seeded into a 24-well plate.

### 4.8. Cytotoxicity Assays

Following the addition of ab112118 (Abcam, Cambridge, U.K.) reagent, HCAECs and HITAECs cultured in 96-well plates (85–90% confluence) were exposed to 10 μL of MPPs, CPP-P, CPP-S (OD_650_ = 0.08–0.10, for CPP-P and CPP-S: equal to 5 μg calcium or 1.2 × 10^4^ OsteoSense 680EX-positive PKH67-negative events), or PBS (n = 24 wells per group) for 4 h with the subsequent calculation of the OD_530_/OD_650_ ratio to evaluate cell proliferation and viability (percent in relation to the vehicle control (PBS) group). The cells were visualised by phase contrast microscopy (AxioObserver.Z1, Carl Zeiss, Stuttgart, Germany) before adding ab112118. Alternatively, HCAECs and HITAECs cultured in 6-well plates (85–90% confluence) were exposed to 100 μL of MPPs, CPP-P, CPP-S (OD_650_ = 0.08–0.10, for CPP-P and CPP-S: equal to 50 μg calcium or 1.2 × 10^5^ OsteoSense 680EX-positive PKH67-negative events), or PBS (n = 11 wells per group) for 4 h with subsequent Hoechst 33342 (2 μg/mL, H3570, Thermo Fisher Scientific, Waltham, MA, USA) and ethidium bromide (10 μg/mL, IB40075, VWR, Radnor, PA, USA) staining for 5 min, washing in a dye-free medium, and fluorescence microscopy (3 fields of view per well, AxioObserver.Z1, Carl Zeiss, Stuttgart, Germany). Quantitative image analysis was performed using ImageJ software (National Institutes of Health, Bethesda, MD, USA).

### 4.9. Internalisation Assays

HCAECs cultured in T-75 flasks (85–90% confluence) were exposed to 1000 μL of CPP-P (OD_650_ = 0.08–0.10, equal to 500 μg calcium or 1.2 × 10^6^ OsteoSense 680EX-positive PKH67-negative events) or PBS for 4 h. The cell pellet was fixed in 2.5% glutaraldehyde (16320, Electron Microscopy Sciences, Hatfield, PA, USA) for 1 h, postfixed in 1% osmium tetroxide for 1 h, embedded into 2% agarose (97062-244, VWR, Radnor, PA, USA), and dehydrated in ascending ethanol series (50%, 60%, 70%, 80%, and 95%, 15 min each) and acetone (1 h). The sections of the agarose gel were then impregnated with an acetone:epoxy resin mixture (1:1) mixture for 2 h and with epoxy resin for 24 h, and were finally embedded into fresh epoxy resin at 60 °C. Ultrafine sections were prepared using an ultramicrotome (LKB Bromma Nova Ultra, LKB, Stockholm, Sweden) placed on a carbon-coated copper grid, then stained with 2% uranyl acetate for 20 min, counterstained with a Reynolds’s lead citrate for 1 h, and examined with a transmission electron microscope.

Alternatively, HCAECs (85–90% confluence) cultured in T-75 flasks (85–90% confluence) were exposed to 1000 μL of MPPs, CPP-P, CPP-S (OD_650_ = 0.08–0.10, for CPP-P and CPP-S: equal to 500 μg calcium or 1.2 × 10^6^ OsteoSense 680EX-positive PKH67-negative events) or PBS for 1 h, and the cell pellet was then treated as described above but without performing ultrafine sectioning. Epoxy resin blocks were then ground, polished, stained with 2% uranyl acetate at 60 °C for 1 h, and counterstained with Reynolds’s lead citrate for 7 min. After a washing in double distilled water, samples were sputter-coated (10 nm thickness) with carbon and visualised by means of backscattered scanning electron microscopy at a 10 kV voltage. The calcium presence in electron-dense dots indicative of internalised CPPs was confirmed by EDX analysis as described above.

To examine whether CPPs enter the lysosomes upon the internalisation, we cultured HCAECs on chambered coverslips (80826, Ibidi, Grafelfing, Germany) to 85–90% confluence, exposed them to 25 μL of fluorescein isothiocyanate (FITC)-labelled CPP-P (OD_650_ = 0.08–0.10, equal to 12.5 μg calcium or 3 × 10^4^ OsteoSense 680EX-positive PKH67-negative events) or PBS for 1 or 4 h, and concurrently stained with LysoTracker Red (500 nmol/L, L7528, Thermo Fisher Scientific, Waltham, MA, USA) for 1 h according to the manufacturer’s protocol. Labeling of CPP-P was performed by their incubation with FITC-labelled albumin (A23015, Thermo Fisher Scientific, Waltham, MA, USA) for 1 h. Nuclear counterstaining was performed by incubation with Hoechst 33342 (2 μg/mL) for 5 min. Visualisation was performed using confocal microscopy (LSM 700, Carl Zeiss, Stuttgart, Germany) after a washing with a dye-free medium.

### 4.10. Lysosome Permeabilisation Assay

HCAECs cultured in 96-well culture plates (85–90% confluence) were exposed to 100 μL of MPPs, CPP-P, CPP-S (OD_650_ = 0.08–0.10, for CPP-P and CPP-S: equal to 50 μg calcium or 1.2 × 10^5^ OsteoSense 680EX-positive PKH67-negative events) or PBS (n = 12 wells per group) for 4 or 24 h with or without specific inhibitor of vacuolar-type H^+^-ATPase bafilomycin A1 (ab120497 (Abcam, Cambridge, UK), 0.1 or 1 μmol/L). Cell proliferation and viability were monitored by a concurrent addition of ab112118 reagent (Abcam, Cambridge, UK) for 4 h and further calculations as described above.

### 4.11. Ca^2+^ Translocation Assay

HCAECs cultured on chambered coverslips (80826, Ibidi, Grafelfing, Germany) to 85–90% confluence were exposed to 25 μL of CPP-P (OD_650_ = 0.08–0.10, equal to 12.5 μg calcium or 3 × 10^4^ OsteoSense 680EX-positive PKH67-negative events) or PBS for 30 min and concurrently stained with a specific Ca^2+^ indicator fluo-3 AM (5 μmol/L, F14218, Thermo Fisher Scientific, Waltham, MA, USA) for 30 min with the subsequent washing with an indicator-free medium and incubation for 30 min to allow complete de-esterification of intracellular AM esters according to the manufacturer’s protocol. Nuclear counterstaining was performed by incubation with Hoechst 33342 (2 μg/mL) for 5 min. Visualisation was performed using confocal microscopy after washing with a dye-free medium.

### 4.12. Assessment of Oxidative Stress

HCAECs cultured on chambered coverslips (80826, Ibidi, Grafelfing, Germany) to 85–90% confluence were exposed to 25 μL of MPPs, CPP-P, CPP-S (OD_650_ = 0.08–0.10, for CPP-P and CPP-S: equal to 12.5 μg calcium or 3 × 10^4^ OsteoSense 680EX-positive PKH67-negative events) or PBS for 1 or 4 h and concurrently stained with MitoSOX Red (5 μmol/L, M36008, Thermo Fisher Scientific, Waltham, MA, USA), a fluorogenic probe for superoxide, for 10 min; or with CellROX Green (5 μmol/L, C10444, Thermo Fisher Scientific, Waltham, MA, USA), a fluorogenic probe for total reactive oxygen species, for 30 min. Nuclear counterstaining was performed by incubation with Hoechst 33342 (2 μg/mL) for 5 min. Visualisation was performed using confocal microscopy after washing with PBS.

Alternatively, HCAECs cultured in 96-well culture plates (85–90% confluence) were exposed to 100 μL of MPPs, CPP-P, CPP-S (OD_650_ = 0.08–0.10, for CPP-P and CPP-S: equal to 50 μg calcium or 1.2 × 10^5^ OsteoSense 680EX-positive PKH67-negative events) or PBS (n = 12 wells per group) for 4 or 24 h with or without antioxidant enzymes superoxide dismutase (SOD, 250 U/mL) and catalase (CAT, 500 U/mL). Both SOD and CAT were added 3 h before exposure to the particles to ensure their internalisation through the plasma membrane. Cell proliferation and viability were monitored by the concurrent addition of ab112118 reagent (Abcam, Cambridge, U.K.) for 4 h and further calculations as described above.

### 4.13. Thiobarbituric Acid Reactive Substances (TBARS) Assay

HCAECs and HITAECs cultured in 6-well plates (85–90% confluence) were exposed to 100 μL of MPPs, CPP-P, CPP-S (OD_650_ = 0.08–0.10, for CPP-P and CPP-S: equal to 50 μg calcium or 1.2 × 10^5^ OsteoSense 680EX-positive PKH67-negative events) or PBS (n = 11 wells per group) for 24 h. Conditioned medium (2 mL per well) was then collected and measured for thiobarbituric acid reactive substances (TBARS), by-products of lipid peroxidation, by the addition of 1 mL of thiobarbituric acid (T5500, Sigma-Aldrich, St. Louis, MO, USA) and 3 mL of H_3_PO_4_ (695017, Sigma-Aldrich, St. Louis, MO, USA) to 250 μL of the sample. After 60 min incubation at 100 °C and cooling at 8 °C for 5 min, 1 mL of 1-butanol (360465, Sigma-Aldrich, St. Louis, MO, USA) was added for the enrichment of the TBARS fraction. Upon 10 min centrifugation at 3000 rpm, the supernatant was aliquoted in 96-well plates (100 μL per well, each sample was aliquoted in triplicate), followed by the measurement of OD_450_. The level of TBARS in each sample was then calculated through the division of OD_450_ by the molar extinction coefficient of malondialdehyde (0.156) and then multiplication by the sample dilution factor (16).

### 4.14. Gene Expression Profiling

After exposure of HCAECs and HITAECs cultured in 6-well plates (85–90% confluence) to 100 μL of MPPs, CPP-P, CPP-S (OD_650_ = 0.08–0.10, for CPP-P and CPP-S: equal to 50 μg calcium or 1.2 × 10^5^ OsteoSense 680EX-positive PKH67-negative events), or PBS (3 wells per group) for 4 h, total RNA was extracted using TRIzol (15596018, Thermo Fisher Scientific, Waltham, MA, USA) and then reverse-transcribed using a High Capacity cDNA Reverse Transcription Kit (4368814, Thermo Fisher Scientific, Waltham, MA, USA). Gene expression was measured by quantitative polymerase chain reaction (qPCR) using customised primers (500 nmol/L each, Evrogen, Moscow, Russia, Table S3), cDNA (20 ng) and PowerUp SYBR Green Master Mix (A25778, Thermo Fisher Scientific, Waltham, MA, USA) according to the manufacturer’s protocol for T_m_ ≥ 60 °C (fast cycling mode). Technical replicates (n = 3 per sample collected from one well) were performed in all qPCR experiments. Quantification of the mRNA levels was performed using the 2^−ΔΔCt^ method. Relative transcript levels are expressed as a value relative to the average of three housekeeping genes (*GAPDH*, *ACTB*, and *B2M*) and to the PBS group (2^−ΔΔCt^).

### 4.15. Cytokine Secretion Profiling

HCAECs and HITAECs cultured in 6-well plates (85–90% confluence) were exposed to 100 μL of MPPs, CPP-P, CPP-S (OD_650_ = 0.08–0.10, for CPP-P and CPP-S: equal to 50 μg calcium or 1.2 × 10^5^ OsteoSense 680EX-positive PKH67-negative events), or PBS for 4 h. Upon the collection of the conditioned medium (300 μL per well), the levels of interleukin-6 and -8 in cell culture supernatant were measured by enzyme-linked immunosorbent assay using the respective kits (ab178013 and ab46032, Abcam, Cambridge, UK) according to the manufacturer’s protocol.

Alternatively, HCAECs and HITAECs cultured in T-75 flasks (85–90% confluence) were exposed to 1000 μL of CPP-P (OD_650_ = 0.08–0.10, equal to 500 μg calcium or 1.2 × 10^6^ OsteoSense 680EX-positive PKH67-negative events) or PBS for 4 h. Upon the collection of the conditioned medium (2 mL per flask) and cell lysis by RIPA buffer (89901, Thermo Fisher Scientific, Waltham, MA, USA) supplemented with protease and phosphatase inhibitors (78444, Thermo Fisher Scientific, Waltham, MA, USA), we profiled the conditioned medium and cell lysate (1 mg protein) for a wide spectrum of cytokines using the respective dot blotting kit (ARY005B, R&D Systems, Minneapolis, MN, USA) according to the manufacturer’s protocol. Additionally, primary human perivascular adipocytes were treated with CPP-P (50 μL of particles per well of a 24-well plate, OD_650_ = 0.08–0.10, equal to 25 μg calcium or 0.6 × 10^5^ OsteoSense 680EX-positive PKH67-negative events) or PBS for 24 h. Conditioned medium (1 mL per well) and cell lysate (200 μg protein) were then collected and profiled for adipokines and cytokines using the respective dot blotting kit (ARY024, R&D Systems, Minneapolis, MN, USA) according to the manufacturer’s protocol. Chemiluminescent detection was performed using a C-DiGit blot scanner (LI-COR Biosciences, Lincoln, NE, USA). Densitometry was performed using ImageJ software (National Institutes of Health, Bethesda, MD, USA).

### 4.16. Leukocyte Adhesion Assay

HCAECs and HITAECs were cultured in flow system chambers (80126, Ibidi, Grafelfing, Germany) to 85–90% confluence, preconditioned by a 15 dyn/cm^2^ laminar flow (10964, Ibidi, Grafelfing, Germany) for 24 h, and then exposed to 500 μL of MPPs, CPP-P, CPP-S (OD_650_ = 0.08–0.10, for CPP-P and CPP-S: equal to 250 μg calcium or 0.6 × 10^6^ OsteoSense 680EX-positive PKH67-negative events), or PBS for 4 h. Primary human peripheral blood mononuclear cells (PBMCs) were isolated from a healthy volunteer using Histopaque-1077 (10771, Sigma-Aldrich, St. Louis, MO, USA) and labelled with a CellTracker Green CMFDA Dye (5 μmol/L, C7025, Thermo Fisher Scientific, Waltham, MA, USA) for 30 min according to the respective manufacturer’s protocols. PBMCs were then added to the flow system (125,000 cells per mL; 1,500,000 cells per flow system unit) for 1 h (1 h before the end of the incubation with the particles). Upon the end of the incubation, nuclear counterstaining was performed (Hoechst 33342, 2 μg/mL, 5 min). Fluorescence was visualised after thorough washing in a dye-free medium (40 fields of view per flow system chamber, AxioObserver.Z1, Carl Zeiss, Stuttgart, Germany). Quantitative image analysis was performed using ImageJ software (National Institutes of Health, Bethesda, MD, USA).

### 4.17. Western Blotting

After exposure of HCAECs and HITAECs (85–90% confluence) to MPPs, CPP-P, CPP-S (1000 μL of particles per T-75 flask, OD_650_ = 0.08–0.10, for CPP-P and CPP-S: equal to 500 μg calcium or 1.2 × 10^6^ OsteoSense 680EX-positive PKH67-negative events), or PBS for 4 h, the total protein was extracted as described above. Equal amounts of protein (16 μg) were mixed with NuPAGE LDS sample buffer in a 4:1 ratio and NuPAGE sample reducing agent at a 10:1 ratio, and then loaded on a 1.5 mm NuPAGE 4–12% Bis-Tris protein gel. The 1:1 mixture of Novex Sharp prestained protein standard (LC5800, Thermo Fisher Scientific, Waltham, MA, USA) and MagicMark XP Western protein standard (LC5602, Thermo Fisher Scientific, Waltham, MA, USA) was loaded as a molecular weight marker. Proteins were separated by SDS-PAGE at 150 V for 90 min. Protein transfer was performed using polyvinylidene difluoride transfer stacks (IB24001, Thermo Fisher Scientific, Waltham, MA, USA) and iBlot 2 Gel Transfer Device (Thermo Fisher Scientific, Waltham, MA, USA) according to the manufacturer’s protocols.

Blots were probed with rabbit antibodies to cleaved caspase-3 (9661, Cell Signaling Technology, Danvers, MA, USA; 1:1000 dilution), poly [ADP-ribose] polymerase 1 (PARP-1, 9542, Cell Signaling Technology, Danvers, MA, USA 1:1000), X-linked inhibitor of apoptosis (XIAP, 2042, Cell Signaling Technology, Danvers, MA, USA; 1:1000), plasminogen activator inhibitor 1 (PAI-1, 11907, Cell Signaling Technology, Danvers, MA, USA; 1:1000), vascular cell adhesion molecule 1 (VCAM1, ab134047, Abcam, Cambridge, U.K.; 1:2000), intercellular cell adhesion molecule 1 (ICAM1, ab109361, Abcam, Cambridge, U.K.; 1:1000), Snail and Slug transcription factors (ab180714, Abcam, Cambridge, U.K.; 1:500), kinase insert domain receptor (KDR, ab39256, Abcam, Cambridge, U.K.; 1:1000), vascular endothelial cadherin (VE-cadherin, 361900, Thermo Fisher Scientific, Waltham, MA, USA 1:100), mouse antibodies to CD31 (ab9498, Abcam, Cambridge, U.K.; 1:1000), N-cadherin (MA515633, Thermo Fisher Scientific, Waltham, MA, USA; 1:500), and histone H3 (3638, Cell Signaling Technology, Danvers, MA, USA; 1:1000), or goat antibody to β-tubulin (ab21057, Abcam, Cambridge, U.K.; 1:1000). Horseradish-peroxidase-conjugated goat anti-rabbit (7074, Cell Signaling Technology, Danvers, MA, USA) or goat anti-mouse secondary antibodies (AP130P, Sigma-Aldrich, St. Louis, MO, USA) were used at 1:200 and 1:1000 dilution, respectively. Incubation with the antibodies was performed using iBind Flex Solution Kit (SLF2020, Thermo Fisher Scientific, Waltham, MA, USA), iBind Flex Cards (SLF2010, Thermo Fisher Scientific, Waltham, MA, USA) and Bind Flex Western Device (Thermo Fisher Scientific, Waltham, MA, USA) according to the manufacturer’s protocols. Chemiluminescent detection was performed using SuperSignal West Pico PLUS chemiluminescent substrate (34580, Thermo Fisher Scientific, Waltham, MA, USA) and a C-DiGit blot scanner (LI-COR Biosciences, Lincoln, NE, USA). Densitometry was performed using ImageJ software (National Institutes of Health, Bethesda, MD, USA).

### 4.18. Statistical Analysis

Statistical analysis was performed using GraphPad Prism 7 (GraphPad Software, San Diego, CA, USA). For descriptive statistics, data are presented as proportion or media, 25th and 75th percentiles, and range. Two independent groups were compared by Pearson’s chi-squared test with Yates’s correction for continuity or by the Mann–Whitney U-test, whereas three or more groups were compared by the Kruskal–Wallis test with post hoc calculation of false discovery rate (FDR) by the two-stage linear step-up procedure of Benjamini, Krieger, and Yekutieli. Calculation of the odds ratio and the respective 95% confidence intervals was conducted employing a MedCalc online calculator (MedCalc Software, Ostend, Belgium). Correlation analysis was performed using Spearman’s rank correlation coefficient. *P*-values, or *q*-values if FDR was applied (*q*-values are the name given to the adjusted *p*-values found using an optimised FDR approach), ≤0.05 were regarded as statistically significant.

## 5. Conclusions

Here, we demonstrated that patients with cerebrovascular disease and coronary artery disease frequently have reduced serum Ca^2+^-binding capacity, resulting in elevated ionised calcium (Ca^2+^) and an increased generation of CPPs (aggregates of calcium, phosphate, and serum proteins). Intravenous administration of these mineral complexes to normolipidemic and normotensive rats induced intimal hyperplasia and adventitial/perivascular inflammation because of promoting endothelial activation and endothelial-to-mesenchymal transition. We propose that the measurement of the CPP count in clinical practice may be useful in cardiovascular risk assessment and prognostication, whereas the therapeutic elimination of these mineral complexes from the blood by Ca^2+^ chelators, Mg^2+^ supplementation, or crystallisation inhibitors might retard atherosclerosis in patients with disturbed mineral homeostasis.

## Data Availability

The data presented in this study are available on request from the corresponding author. The data are not publicly available due to privacy restrictions.

## Supplementary Materials

The following are available online at www.mdpi.com/1422-0067/222/21/2458/s1.

## Abbreviations

CCL2—Chemokine (C-C motif) ligand 2, CKD—chronic kidney disease, CPPs—Calciprotein particles, CPP-P—Primary calciprotein particles, CPP-S—Secondary calciprotein particles, CVD—Cardiovascular disease, CXCL1—Chemokine (C-X-C motif) ligand 1, ECs—Endothelial cells, ESRD—End-stage renal disease, ICAM1—Intercellular cell adhesion molecule 1, IL—Interleukin, MCs—Macrophage clusters, MIF—Macrophage migration inhibitory factor, MPPs—Magnesiprotein particles, OD—Optical density, PAI-1—Plasminogen activator inhibitor 1, PARP-1—Poly [ADP-ribose] polymerase 1, PBS—Phosphate-buffered saline, SCARF1—Scavenger receptor class F member 1, SELE—E-selectin, SELP—P-selectin, SNAI—Snail family transcriptional repressor 1, TWIST1—Twist family BHLH transcription factor 1, VLDLR—very low-density lipoprotein receptor, VV—Vasa vasorum, XIAP—X-linked inhibitor of apoptosis protein, ZEB1—Zinc finger E-box-binding homeobox 1.
