# Supplementary material for: Calciprotein Particles Link Disturbed Mineral Homeostasis with Cardiovascular Disease by Causing Endothelial Dysfunction and Vascular Inflammation
Source: Int J Mol Sci. 2021 Nov 18;22(22):12458. doi: 10.3390/ijms222212458 (PMC8626027; doi:10.3390/ijms222212458)
Supplement: Supplementary file 1 [file ijms-22-12458-s001.zip › ijms-1436100-supplementary 2.pdf]

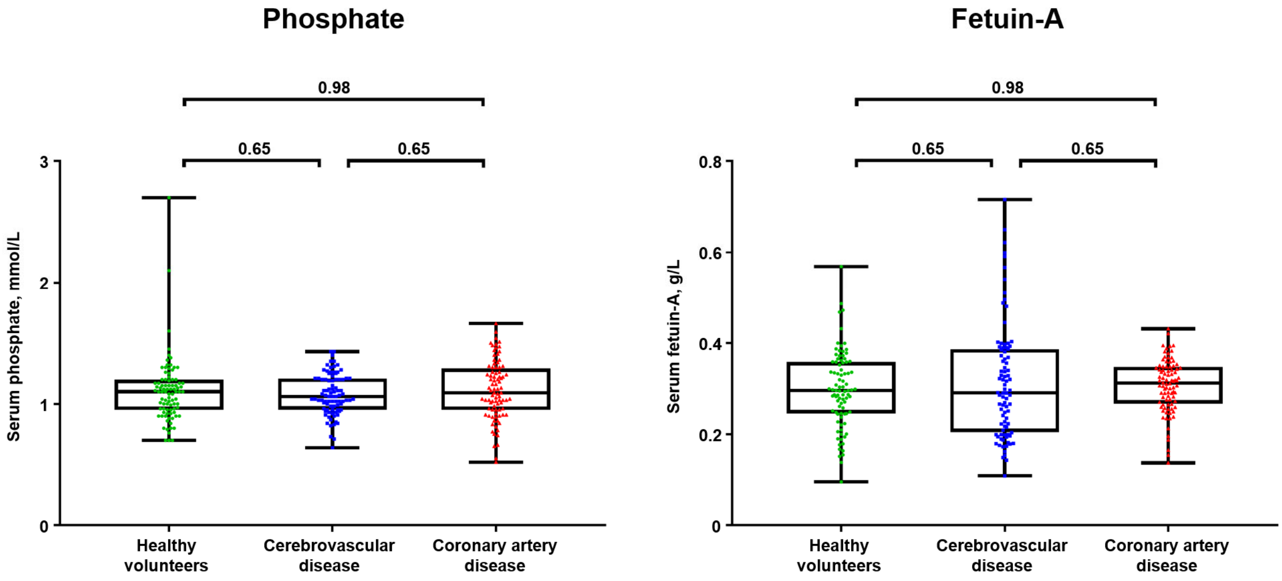

Figure S1. **Patients with cerebrovascular disease or coronary artery disease are not characterised by altered serum phosphate and fetuin-A levels.** Each dot represents a serum sample collected from one subject (n = 44 per group). Whiskers indicate range, boxes bounds indicate 25th–75th percentiles, center lines indicate median. P values provided above boxes, Kruskal-Wallis test with post hoc false discovery rate correction by two-stage linear step-up procedure of Benjamini, Krieger and Yekutieli.

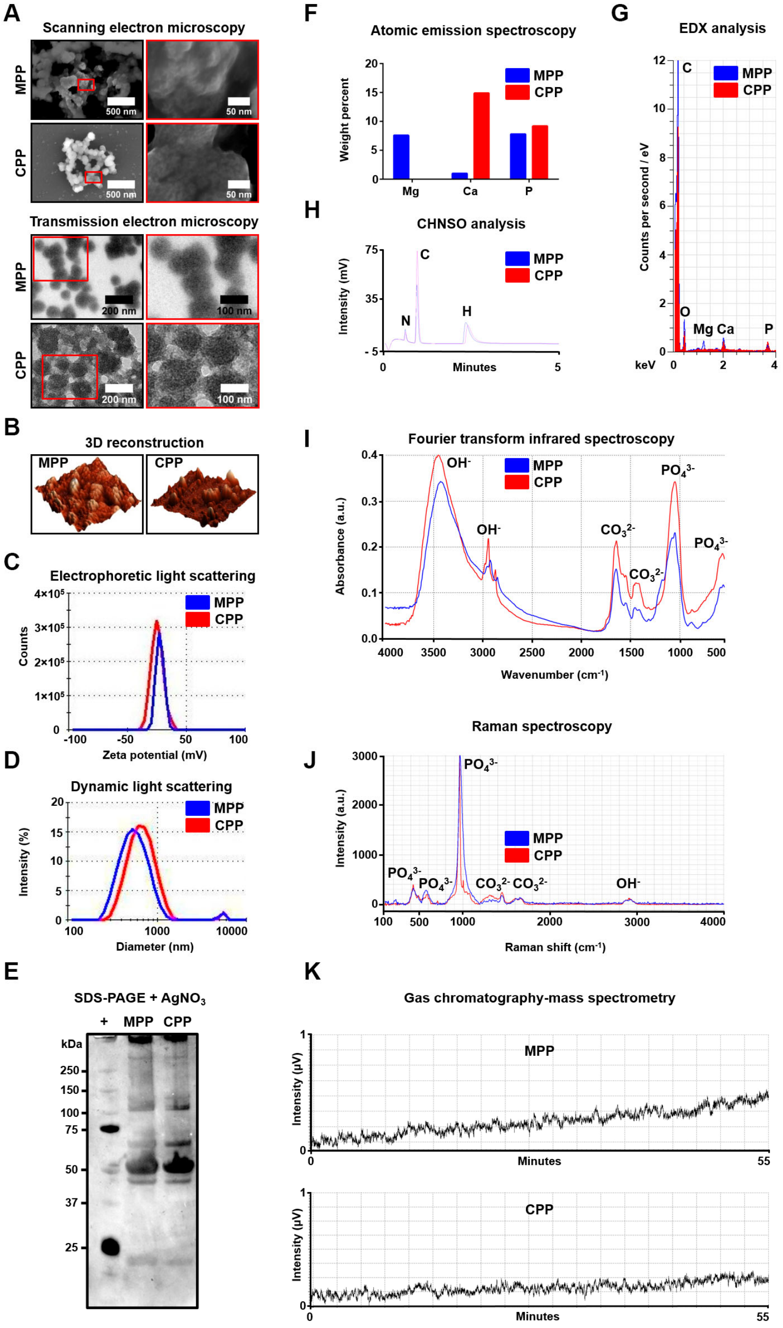

Figure S2. ***In vitro*** **synthesised MPPs and CPPs display an indistinguishable morphology, particle-size distribution, mineral and organic patterns excepting the magnesium/calcium content.** **(a)** Scanning and transmission electron microscopy images showing that both MPPs and CPPs are morphologically indistinguishable spherical particles; **(b)** 3D reconstruction of MPPs and CPPs by atomic force microscopy; **(c)** Electrophoretic light scattering plot shows that MPPs and CPPs have a nearly identical surface charge (from -22 to -25 mV); **(d)** Dynamic light scattering measurements demonstrate that MPPs and CPPs have a similar particle-size distribution curve, with a diameter range of 100–1000 nm and average diameter of around 500 nm; **(e)** Sodium dodecyl sulphate-polyacrylamide gel electrophoresis with the subsequent silver staining show that MPPs and CPPs have a similar protein profile; **(f)** Atomic emission spectroscopy confirms that MPPs primarily consist from magnesium and phosphorus whereas CPPs contain calcium and phosphorus; **(g)** Energy-dispersive X-ray spectroscopy shows the presence of carbon, oxygen, calcium, and phosphorus in both MPPs and CPPs whilst MPPs additionally contain magnesium; **(h)** CHNSO analysis allows detection of nitrogen, carbon, and hydrogen in both MPPs and CPPs; **(i)** Fourier transform infrared spectroscopy identifies phosphate (PO_4^{3-}), carbonate (CO_3^{2-}), and hydroxyl (OH^-) groups in both MPPs and CPPs; **(j)** Raman spectroscopy verifies findings obtained by Fourier transform infrared spectroscopy; **(k)** Gas chromatography-mass spectrometry reveals an absence of lipids in MPPs and CPPs. MPPs – magnesiprotein particles, CPPs – calciprotein particles, EDX – energy-dispersive X-ray spectroscopy.

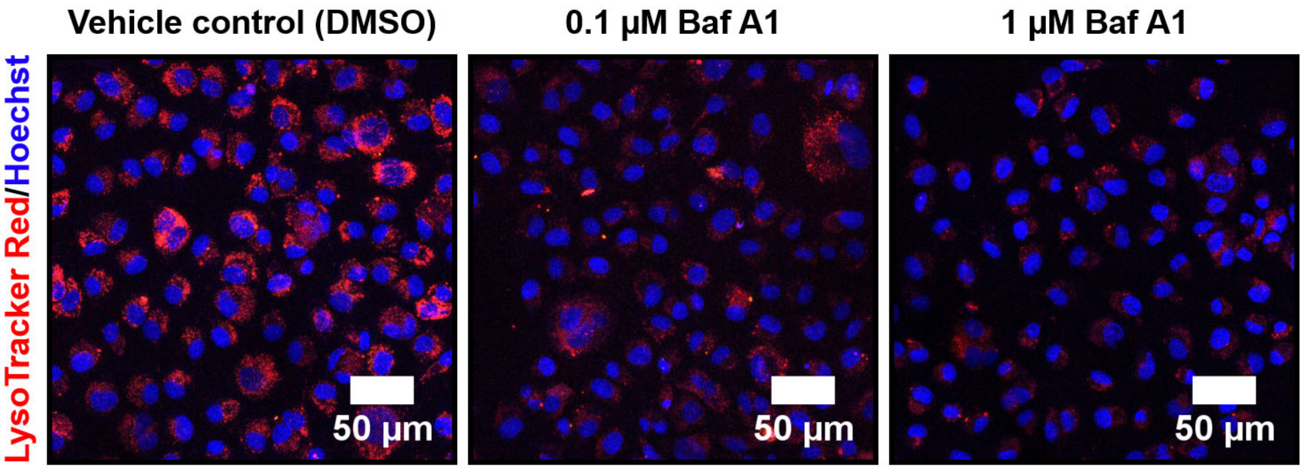

Figure S3. Bafilomycin A1 increases lysosomal pH in endothelial cells. Primary human coronary artery endothelial cells were incubated with ascending concentrations of bafilomycin A1 or vehicle control for 4 hours and stained with Hoechst 33342 (nuclear stain, blue colour) and LysoTracker Red (lysosomes, red colour). Note the reduction of red staining along with the increasing dose of bafilomycin A1. Baf A1 – bafilomycin A1.

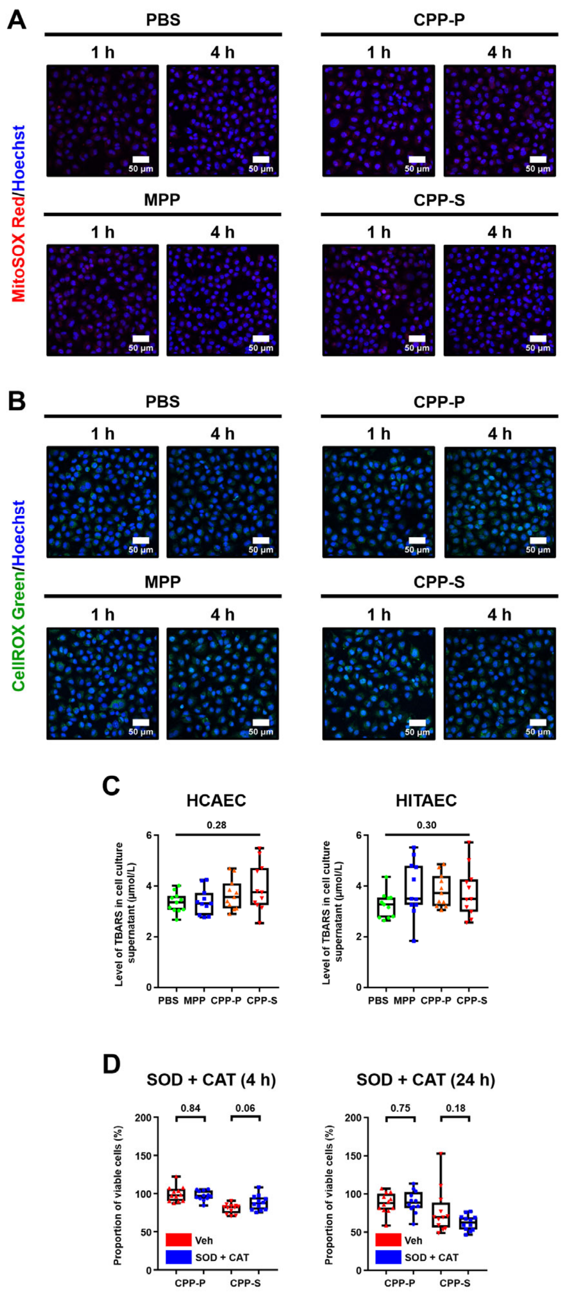

Figure S4. Oxidative stress is not among the major CPP effects on endothelial cells. (a) HCAEC and HITAEC were cultured in the presence of PBS, MPPs, CPP-P or CPP-S (25 μL of particles per live cell imaging culture chamber, OD_{650} = 0.08-0.10) for 1 or 4 hours with the subsequent incubation with MitoSOX Red, a fluorogenic probe for superoxide. Representative images; **(b)** Similar experimental setup employing CellROX Green, a fluorogenic probe for total reactive oxygen species. Representative images; **(c)** HCAEC and HITAEC were cultured in the presence of PBS, MPPs, CPP-P or CPP-S (100 μL of particles per well of 6-well plate, OD_{650} = 0.08-0.10) for 24 hours (n = 11 wells per group). Conditioned media was collected and profiled for thiobarbituric acid reactive substances (TBARS) which represent by-products of lipid peroxidation. Each dot represents one well of the culture plate. Whiskers indicate range, boxes bounds indicate 25th–75th percentiles, center lines indicate median. *P* values provided above boxes, Kruskal-Wallis test; **(d)** HCAEC and HITAEC were cultured in the presence of PBS, MPPs, CPP-P or CPP-S (10 μL of particles per well of 96-well plate, OD_{650} = 0.08-0.10) for 4 or 24 hours (n = 12 wells per group). Concurrent addition of superoxide dismutase (SOD, 250 U/mL) and catalase (CAT, 500 U/mL) did not rescue HCAEC from CPP-induced death regardless of exposure time. Each dot represents one well of the culture plate. Whiskers indicate range, boxes bounds indicate 25th–75th percentiles, center lines indicate median. *P* values provided above boxes, Mann-Whitney U-test. HCAEC – human coronary artery endothelial cells, HITAEC – human internal thoracic artery endothelial cells, PBS – phosphate-buffered saline, MPPs – magnesiprotein particles, CPP-P – primary calciprotein particles, CPP-S – secondary calciprotein particles, TBARS – thiobarbituric acid reactive substances, SOD – superoxide dismutase, CAT – catalase.

Table S1. Correlation between the mineral homeostasis parameters in all study participants (n = 264, Spearman's rank correlation coefficient). Statistically significant correlations are marked bold.

	Ionised calcium	Total protein	Albumin	Phosphate	Fetuin-A	OD_{650}
**Ionised calcium**		-0.140	-0.150	0.112	-0.066	0.054
**Total protein**	-0.140		**0.869**	0.233	0.111	**-0.315**
**Albumin**	-0.150	**0.869**		0.140	0.134	**-0.298**
**Phosphate**	0.112	0.233	0.140		0.132	-0.006
**Fetuin-A**	-0.066	0.111	0.134	0.132		0.002
**OD_{650}**	0.054	**-0.315**	**-0.298**	-0.006	0.002	

Table S2. Clinicopathological features of the study participants. Statistically significant associations are marked bold. Data are presented as proportions or median with interquartile range. Pearson's chi-squared test with Yates's correction for continuity or Mann-Whitney U-test.

Patient group / Confounder	Brain ischemia (n = 44)	Ischemic stroke (n = 44)	*P*	Stable angina (n = 44)	Myocardial infarction (n = 44)	*P*	Cerebrovascular disease (n = 88)	Coronary artery disease (n = 88)	*P*	Cardiovascular disease (n = 176)	Healthy blood donors (n = 88)	*P*
**Age and gender**												
Male gender	26/44 (59.1%)	31/44 (70.5%)	0.37	35/44 (79.5%)	30/44 (68.2%)	0.33	57/88 (64.8%)	65/88 (73.9%)	0.25	122/176 (69.3%)	51/88 (58.0%)	0.09
Age, years	67.0 (61.0-73.7)	64.50 (59.2-70.0)	0.07	64.0 (58.0-69.0)	63.0 (56.7-71.7)	0.95	66.0 (61.0-72.0)	64.0 (58.0-69.0)	0.09	65.0 (60.0-71.0)	63.0 (57.2-67.0)	**0.01**
**Comorbid conditions**												
Arterial hypertension	41/43 (95.3%)	39/41 (95.1%)	0.64	38/42 (90.5%)	26/31 (83.9%)	0.62	80/84 (95.2%)	64/73 (87.7%)	0.15	144/157 (91.7%)	31/88 (35.2%)	**0.01**

Chronic heart failure	36/43 (83.7%)	37/41 (90.2%)	0.57	41/42 (97.6%)	12/31 (38.7%)	**0.01**	73/84 (86.9%)	53/73 (72.6%)	**0.04**	126/157 (80.2%)	3/88 (3.4%)	**0.01**
Chronic obstructive pulmonary disease or asthma	3/43 (7.0%)	7/41 (17.1%)	0.27	0/42 (0.0%)	4/31 (12.9%)	0.06	10/84 (11.9%)	4/73 (5.5%)	0.26	14/157 (8.9%)	11/88 (12.5%)	0.50
Smoking	2/43 (4.6%)	6/41 (14.6%)	0.24	18/42 (42.9%)	20/31 (64.5%)	0.11	8/84 (9.5%)	38/73 (52.0%)	**0.01**	46/157 (29.3%)	22/88 (25.0%)	0.57
Chronic kidney disease	4/43 (9.3%)	4/41 (9.8%)	0.76	9/42 (21.4%)	3/31 (9.7%)	0.31	8/84 (9.5%)	12/73 (16.4%)	0.29	20/157 (12.7%)	6/88 (6.8%)	0.22
Diabetes mellitus	10/43 (23.2%)	13/41 (31.7%)	0.53	12/42 (28.6%)	6/31 (19.4%)	0.53	23/84 (27.4%)	18/73 (24.7%)	0.84	41/157 (26.1%)	3/88 (3.4%)	**0.01**
Overweight	25/43 (58.1%)	20/41 (48.8%)	0.52	18/42 (42.9%)	3/31 (9.7%)	**0.01**	45/84 (53.6%)	21/73 (28.8%)	**0.01**	66/157 (42.0%)	30/88 (34.1%)	0.28
Obesity	5/43 (11.6%)	9/41 (22.0%)	0.33	16/42 (38.1%)	14/31 (45.2%)	0.71	14/84 (16.7%)	30/73 (41.0%)	**0.01**	44/157 (28.0%)	40/88 (45.4%)	**0.01**
**Quantitative parameters**												
Body mass index, kg/m²	27.6 (24.2-32.0)	26.3 (24.6-32.8)	0.88	28.71 (25.9-31.8)	27.0 (23.0-33.0)	0.29	26.9 (24.5-32.1)	28.5 (24.4-32.3)	0.81	27.8 (24.5-32.2)	28.8 (25.3-32.4)	0.25
Glomerular filtration rate, mL/min/1.73 m²	73.0 (60.0-82.0)	77.0 (66.0-91.5)	0.13	87.0 (70.7-96.5)	84.0 (65.0-96.0)	0.67	74.0 (63.2-87.7)	86.0 (66.5-96.0)	**0.01**	78.0 (65.0-92.0)	90.0 (77.0-98.0)	**0.01**
Left ventricular ejection fraction, %	64.0 (60.5-65.5)	65.0 (64.0-67.0)	0.10	62.0 (48.0-65.0)	52.0 (45.0-61.0)	**0.01**	64.0 (62.0-65.7)	55.0 (46.0-63.0)	**0.01**	62.0 (52.5-65.0)	-	-
Number of affected coronary arteries	-	-		3.0 (2.0-3.0)	2.0 (1.0-3.0)	**0.01**	-	2.0 (2.0-3.0)		-	-	-
Extracranial artery stenosis percent	75.0 (70.0-83.5)	86.0 (75.5-95.0)	**0.01**	-	-	-	80.0 (70.0-90.0)	-	-	-	-	-
**Pre-treatment**												

Antiplatelet drugs	25/43 (58.1%)	15/41 (36.6%)	0.08	33/42 (78.6%)	16/31 (51.6%)	**0.03**	40/84 (47.6%)	49/73 (67.1%)	**0.02**	89/157 (56.7%)	15/88 (17.0%)	**0.01**
Beta blockers	16/43 (37.2%)	13/41 (31.7%)	0.76	28/42 (66.7%)	14/31 (45.2%)	0.11	29/84 (34.5%)	42/73 (57.5%)	**0.01**	71/157 (45.2%)	7/88 (8.0%)	**0.01**
Angiotensin-converting enzyme inhibitors	7/43 (16.3%)	3/41 (7.3%)	0.35	17/42 (40.5%)	11/31 (35.5%)	0.85	10/84 (11.9%)	28/73 (38.4%)	**0.01**	38/157 (24.2%)	13/88 (14.8%)	0.11
Statins	29/43 (67.4%)	14/41 (34.1%)	**0.01**	35/42 (83.3%)	18/31 (58.1%)	**0.03**	43/84 (51.2%)	53/73 (72.6%)	**0.01**	96/157 (61.1%)	4/88 (4.5%)	**0.01**
Nitrates	0/43 (0.0%)	0/41 (0.0%)	0.91	2/42 (4.8%)	0/31 (0.0%)	0.61	0/84 (0.0%)	2/73 (2.7%)	0.41	2/157 (1.3%)	0/88 (0.0%)	0.75
Angiotensin receptor II blockers	16/43 (37.2%)	5/41 (12.2%)	**0.02**	16/42 (38.1%)	8/31 (25.8%)	0.39	21/84 (25.0%)	24/73 (32.9%)	0.36	45/157 (28.7%)	10/88 (11.4%)	**0.01**
Aldosterone antagonists	2/43 (4.7%)	1/41 (2.4%)	0.96	3/42 (7.1%)	1/31 (3.2%)	0.83	3/84 (3.6%)	4/73 (5.5%)	0.85	7/157 (4.5%)	0/88 (0.0%)	0.11
Calcium channel blockers	14/43 (32.5%)	5/41 (12.2%)	0.05	19/42 (45.2%)	9/31 (29.0%)	0.24	19/84 (22.6%)	28/73 (38.4%)	0.05	47/157 (29.9%)	7/88 (8.0%)	**0.01**
Diuretics	0/43 (0.0%)	1/41 (2.4%)	0.97	7/42 (16.7%)	4/31 (12.9%)	0.91	1/84 (1.2%)	11/73 (15.1%)	**0.01**	12/157 (7.6%)	6/88 (6.8%)	0.99
Anticoagulants	5/43 (11.6%)	1/41 (2.4%)	0.23	3/42 (7.1%)	1/31 (3.2%)	0.83	6/84 (7.1%)	4/73 (5.5%)	0.92	10/157 (6.4%)	1/88 (1.1%)	0.12

Table S3. Primers for qPCR and parameters of the reaction.

Gene	Primers	T_m, °C	R^2	Eff, %
ACTB	F: 5′-CATCGAGCACGGCATCGTCA-3′<br>R: 5′-TAGCACAGCCTGGACAGCAAC-3′	≥ 60	0.998	91.3
GAPDH	F: 5′-AGCCACATCGCTCAGACAC-3′<br>R: 5′-GCCCAATACGACCAAATCC-3′	≥ 60	0.993	94.4
B2M	F: 5′-TCCATCCGACATTGAAGTTG-3′<br>R: 5′-CGGCAGGCATACTCATCTT-3′	≥ 60	0.988	100.8
IL6	F: 5′-GGCACTGGCAGAAAACAACC-3′<br>R: 5′-GCAAGTCTCCTCATTGAATCC-3′	≥ 60	0.997	106.9
CXCL8	F: 5′-CAGAGACAGCAGAGCACAC-3′<br>R: 5′-AGTTCTTTAGCACTCCTTGGC-3′	≥ 60	0.981	102.5
CCL2	F: 5′-TTCTGTGCCTGCTGCTCATAG-3′<br>R: 5′-AGGTGACTGGGGCATTGATTG-3′	≥ 60	0.996	63.7
IL12A	F: 5′-GCCTTCACCACTCCCAAAAC-3′<br>R: 5′-TGTCTGGCCTTCTGGAGCAT-3′	≥ 60	0.996	81.9
IL1R1	F: 5′-GGCTGAAAAGCATAGAGGGAAC-3′<br>R: 5′-CTGGGCTCACAATCACAGG-3′	≥ 60	0.998	101.9
SNAI1	F: 5′-CAGACCCACTCAGATGTCAAGAA-3′<br>R: 5′-GGGCAGGTATGGAGAGGAAGA-3′	≥ 60	0.983	98.7
SNAI2	F: 5′-ACTCCGAAGCCAAATGACAA-3′<br>R: 5′-CTCTCTCTGTGGGTGTGTGT-3′	≥ 60	0.986	71.9
TWIST1	F: 5′-GTCCGCAGTCTTACGAGGAG-3′<br>R: 5′-GCTTGAGGGTCTGAATCTTGCT-3′	≥ 60	0.996	73.0
ZEB1	F: 5′-GATGATGAATGCGAGTCAGATGC-3′<br>R: 5′-ACAGCAGTGTCTTGTTGTTGT-3′	≥ 60	0.992	109.4
VLDLR	F: 5′-AGAAAAGCCAAATGTGAACCCT-3′<br>R: 5′-CACTGCCGTCAACACAGTCT-3′	≥ 60	0.992	96.2
SCARF1	F: 5′-CCGATCAGACCTCAAGGACAG-3′<br>R: 5′-CCCAGGGTAGCTTGTGGGA-3′	≥ 60	0.982	104.0
ICAM1	F: 5′-TTGGGCATAGAGACCCCGTT-3′<br>R: 5′-GCACATTGCTCAGTTCATACACC-3′	≥ 60	0.994	95.0
SELE	F: 5′-GCACAGCCTTGTCCAACC-3′<br>R: 5′-ACCTCACCAAACCCTTCG-3′	≥ 60	0.982	104.2

SELP	F: 5′-ATGGGTGGGAACCAAAAAGG-3′<br>R: 5′-GGCTGACGGACTCTTGATGTAT-3′	≥ 60	0.992	80.7
VIM	F: 5′-CGCCAGATGCGTGAAATGG-3′<br>R: 5′-ACCAGAGGGAGTGAATCCAGA-3′	≥ 60	0.990	100.5
MMP2	F: 5′-CCGTGTTTGCCATCTGTTTTAG-3′<br>R: 5′-AGGTTCTCTTGCTGTTTACTTTGGA-3′	≥ 60	0.993	86.4
CDH2	F: 5′-GCTTCTGGTGAAATCGCATTA-3′<br>R: 5′-AGTCTCTCTTCTGCCTTTGTAG-3′	≥ 60	0.968	102.4
KDR	F: 5′-TGCCTACCTCACCTGTTTC-3′<br>R: 5′-GGCTCTTTCGCTTACTGTTC-3′	≥ 60	0.996	90.8
TNFRSF1A	F: 5′-CCAGGAGAAACAGAACACCGT-3′<br>R: 5′-AAACCAATGAAGAGGAGGGATAA-3′	≥ 60	0.99	106.7
ACTA2	F: 5′-GTGTTGCCCCTGAAGAGCAT-3′<br>R: 5′-GCTGGGACATTGAAAGTCTCA-3′	≥ 60	0.985	105.3
VCAM1	F: 5′-CGTCTTGGTCAGCCCTTCCT-3′<br>R: 5′-ACATTCATATACTCCCGCATCCTTC-3′	≥ 60	0.969	68.3
SMTN	F: 5′-GGGATCGTGTCCACAAGTTCA-3′<br>R: 5′-GCTACTCCTCGTTGCTCCTT-3′	≥ 60	0.984	77.4
LDLR	F: 5′-ACGGCGTCTCTTCCTATGACA-3′<br>R: 5′-CCCTTGGTATCCGCAACAGA-3′	≥ 60	0.99	92.3
CDH5	F: 5′-AAGCGTGAGTCGCAAGAATG-3′<br>R: 5′-TCTCCAGGTTTTCGCCAGTG-3′	≥ 60	0.984	104.3
vWF	F: 5′-CCTTGACCTCGGACCCTTATG-3′<br>R: 5′-GATGCCCGTTCACACCACT-3′	≥ 60	0.984	100.2
PXDN	F: 5′-AGCCAGCCATCACCTGGAAC-3′<br>R: 5′-TTCCGGGCCACACACTCATA-3′	≥ 60	0.988	94.6
TNFRSF1B	F: 5′-GTCCACACGATCCCAACAC-3′<br>R: 5′-CACACCCACAATCAGTCCAA-3′	≥ 60	0.984	106.8
NOS3	F: 5′-GTGATGGCGAAGCGAGTGAAG-3′<br>R: 5′-CCGAGCCCGAACACACAGAAC-3′	≥ 60	0.985	85.2
COL4A1	F: 5′-GGACTACCTGGAACAAAAGGG-3′<br>R: 5′-GCCAAGTATCTCACCTGGATCA-3′	≥ 60	0.998	105.9
